# Early prediction of macrocrack location in concrete, rocks and other granular composite materials

**DOI:** 10.1038/s41598-020-76616-y

**Published:** 2020-11-20

**Authors:** Antoinette Tordesillas, Sanath Kahagalage, Charl Ras, Michał Nitka, Jacek Tejchman

**Affiliations:** 1 School of Mathematics and Statistics, The University of Melbourne, Melbourne, 3010 Australia; 2 Faculty of Civil and Environmental Engineering, Gdańsk University of Technology, Narutowicza 11/12, 80-233 Gdańsk, Poland

**Keywords:** Engineering, Materials science

## Abstract

Heterogeneous quasibrittle composites like concrete, ceramics and rocks comprise grains held together by bonds. The question on whether or not the path of the crack that leads to failure can be predicted from known microstructural features, viz. bond connectivity, size, fracture surface energy and strength, remains open. Many fracture criteria exist. The most widely used are based on a postulated stress and/or energy extremal. Since force and energy share common transmission paths, their flow bottleneck may be the precursory failure mechanism to reconcile these optimality criteria in one unified framework. We explore this in the framework of network flow theory, using microstructural data from 3D discrete element models of concrete under uniaxial tension. We find the force and energy bottlenecks emerge in the same path and provide an early and accurate prediction of the ultimate macrocrack path ${\mathcal {C}}$. Relative to all feasible crack paths, the Griffith’s fracture surface energy and the Francfort–Marigo energy functional are minimum in ${\mathcal {C}}$; likewise for the critical strain energy density if bonds are uniformly sized. Redundancies in transmission paths govern prefailure dynamics, and predispose ${\mathcal {C}}$ to cascading failure during which the concomitant energy release rate and normal (Rankine) stress become maximum along ${\mathcal {C}}$.

## Introduction

Force and strain energy share common pathways for flow. For many everyday quasibrittle materials like concrete, ceramics, asphalt mixtures, and rocks, these pathways are embodied in a disordered network of intergranular bonds with different geometric and constitutive properties^1–4^. While there is broad recognition that the bulk strength and fracture of granular composites are governed by subscale heterogeneities, the details of these dependencies remain mired in controversy^1,5,6^. Many engineering challenges depend on a detailed understanding of these microstructural-to-bulk relationships. In the built environment, the growth in demand for more resilient and sustainable materials continues to outpace development^7^. Although only 2–3% of the Earth’s landmasses, cities consume 60–80% of its energy, produce more than 75% of its greenhouse gases, and consume materials at a level that is projected to reach 90 billion tonnes by 2050^8^. These trends have seen a push for technologies that can harness disorder and heterogeneities in rational design and engineering of high performance and green materials^3,4,6,9–11^. Great impetus for improvements in early detection of damage in materials and structures has also arisen, with the confluence of reduced cost and advances in nondestructive microstructural sensing technology, the rapid growth in construction and manufacturing activities, and the emergence of continuous structural health monitoring to meet government regulations on new and aging infrastructure^12,13^. These challenges have prompted calls for a more nuanced complex systems view of materials, which couple experimental observations with data-driven modeling and simulation, especially in the archetypal composite granular material like concrete^7^. Here, on the centennial anniversary of the founding Griffith’s theory for fracture^14^, we do so in a manner complementary to traditional fracture mechanics.

Specifically, this study seeks to answer: *Can the path of the crack that leads to failure be predicted from the connectivity of transmission paths and their microstructural fracture properties and, if so, how far in advance?* To achieve this, we break with the tradition of modeling fracture propagation and attendant energy and force transmission as flows in a continuum^1,6,15^. Instead, we model these as *flows in a network*^15–20^. To the best of our knowledge, this is the first study that: (i) delivers a method for early and accurate prediction of the ultimate macrocrack location from known microstructural bond properties (i.e., fracture strength and surface energy, size), disorder in the network connectivity (an effect of packing and grain size distribution), and stage of loading; and (ii) reconciles and consolidates, in a single framework, the precursory failure mechanism of energy and force flow bottlenecks with widely used fracture criteria. With this in mind, this study may help generalize fracture mechanics theory to complex materials where discreteness, disorder, path redundancy and heterogeneity are not only the norm rather than the exception—but are the salient features that govern bulk strength and failure.

The approach of modeling energy and force transmission as flows in a network holds several advantages. First it lends well to proven tools for modeling and characterization of transmission dynamics germane to capacitated complex networks (e.g., power grid, telecommunication and road networks etc.)^21,22^. In such networks, each component (e.g., link) has a finite capacity for flow, and the most vulnerable part of the network to cascading failure is the so-called bottleneck where congestion occurs. Flow bottlenecks are a fundamental concept of network resilience and have been considered as precursors for cascading failure in various cyber, transport and infrastructure systems^23^. Prior evidence also demonstrate that these emergent structures can reliably predict, early in the prefailure regime, the ultimate locale of cascading failure for various natural and synthetic granular media^16,17–20^. Second, the problem of finding where the flow bottleneck emerges and how this path is influenced by heterogeneities in transmission pathways can be expressed in terms of an optimization problem. Consequently, this opens up the opportunity to consolidate network flow approaches with widely used fracture mechanics criteria, which are similarly couched in terms of a postulated energy or stress extrema. Third, as shown recently in the work of Patel et al.^24^ on complex biostructures and van der Linden et al.^17^ on porous granular media flow, synergies between continuum finite element methods and discrete network flow analysis can produce insights on subscale heterogeneities not otherwise accessible from a purely continuum mechanics approach.

**Figure 1:**
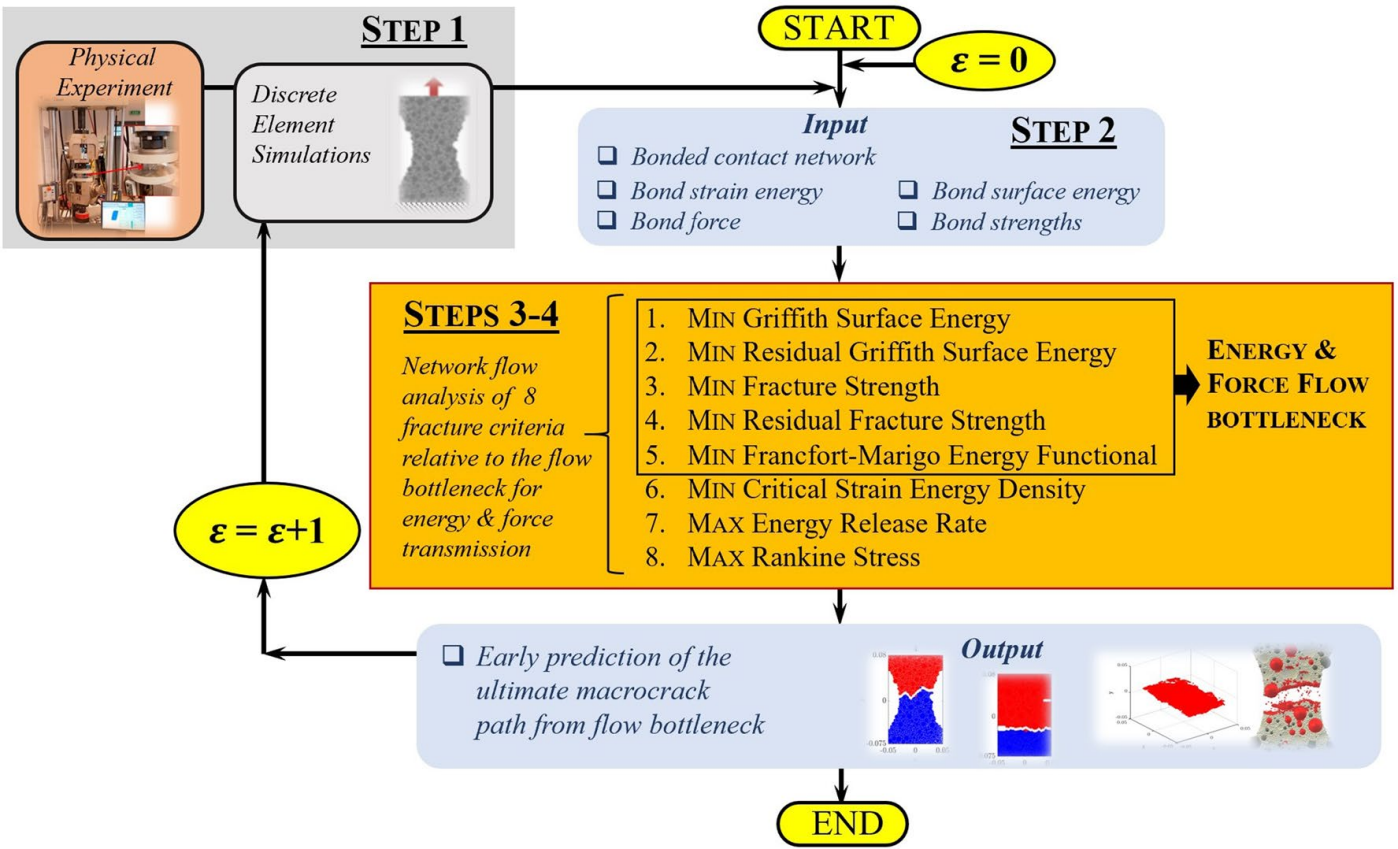
(Color online) Flow chart summarizing the 4-step data-driven analysis of energy and force transmission for early prediction of the ultimate macrocrack path ${\mathcal {C}}$. A prediction of ${\mathcal {C}}$ from each of the eight criteria is generated at each stage of loading $\epsilon =1,2,\ldots ,m$. The path of least resistance to fracture, as given by the flow bottleneck for energy (Criterion 1), force ( Criterion 3), or their respective residuals (Criteria 2, 4), provides an early prediction of ${\mathcal {C}}$.

Heterogeneity in transmission pathways has major implications for the spread of failure in a broad range of networked systems^21,22^. In quasibrittle granular composites, this was recently investigated with respect to the coupled evolution of force and damage propagation in the framework of network flow theory^16^. It showed that the majority of force chains develop in the most direct (shortest possible) routes for force transmission, while the ultimate macrocrack emerged in the recurrent force bottleneck that persisted from the nascent stages of the prefailure regime. Different from the work in^16^, here we study the general case of energy, force and their residual transmission processes; the endmost deals with the proximity to breakage of bonds, viz. the amount of energy or force flow that each bond could still transmit given its capacity and current flow.

**Figure 2:**
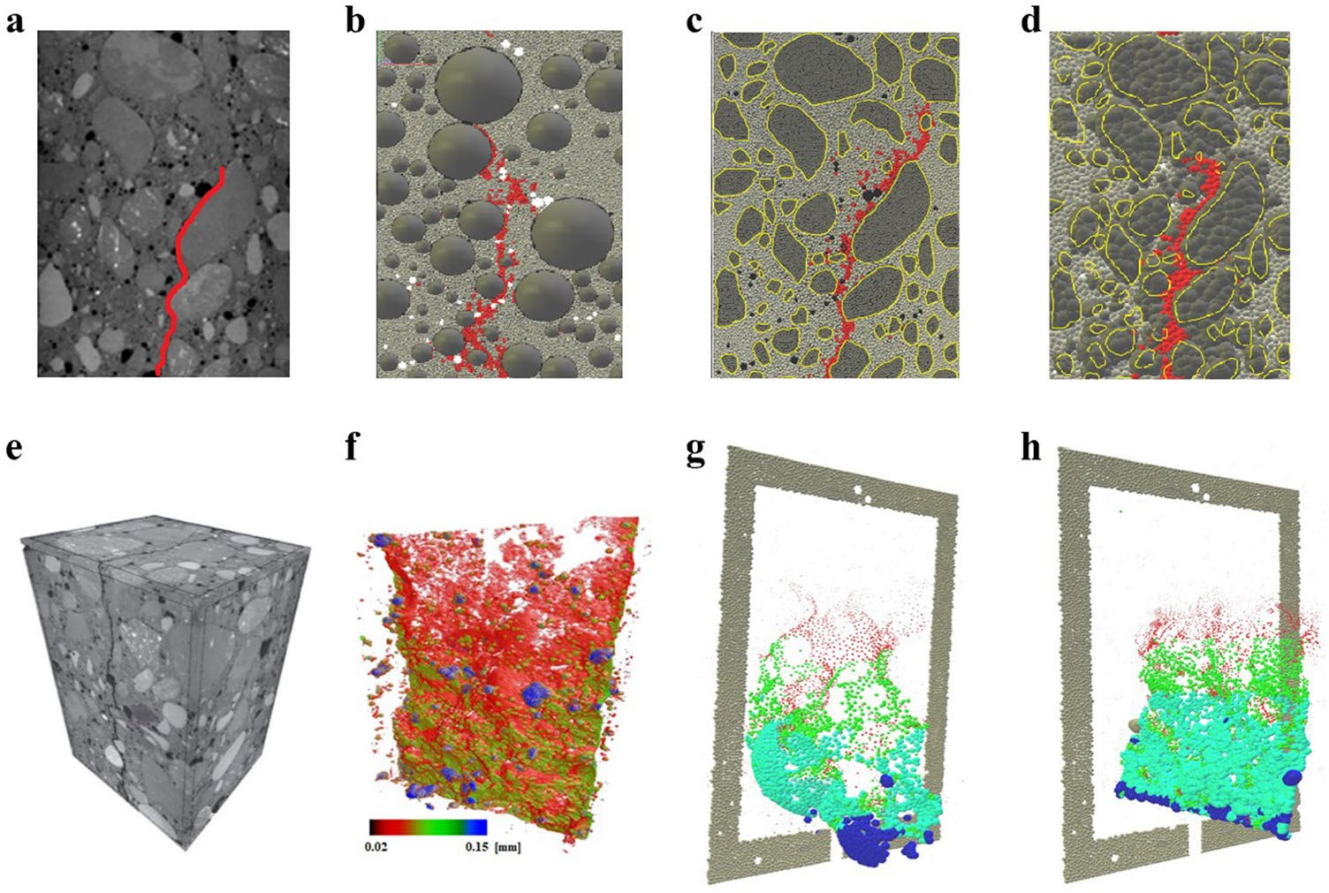
(Color online) The ultimate macrocrack ${\mathcal {C}}$ in a three-point bending test on a concrete beam above the notch at crack-mouth opening displacement (CMOD = 0.10 mm). (**a**) 2D cross sections of the $\mu$CT-image at depth of 3 mm from beam face side: macro-voids (macrocrack) are shown in black (red). (**b**) Planar DEM model with the spherical aggregates and the corresponding real aggregates in the same positions. (**c**) Planar DEM and (**d**) 3D DEM with the aggregates having the same shapes as the real aggregates. (**e**) Full 3D $\mu$CT-image and (**f**) real macrocrack. 3D DEM with (**g**) the spherical aggregates in the same position, and (**h**) the aggregates having the same shapes as the real aggregates.

We proceed in four steps: measure, summarize, predict and characterize (Fig. 1). In Step 1, the input data to the analysis is obtained by measuring the microstructural properties of various samples at distinct stages of a uniaxial tension test. This is achieved through a combined DEM simulation and experimental test campaign which builds on prior investigations on fracture in concrete^24–30^(see also Supplementary file). 3D DEM simulations of real physical tests on concrete were conducted, incorporating the real aggregate size and shape distribution, positions and volume (Fig. 2); these included uniaxial compression and tension, three-point bending and four-point bending tests to ensure the simulations can reproduce realistic quasibrittle fracture patterns in random heterogeneous 3-phase materials composed of aggregate particles, cement matrix and interfacial transitional zones (ITZs), as well as in 2-phase cement composites with no ITZs. In this study, for simplicity, we use data from uniaxial tension test simulations where the particles (aggregate and mortar) are spheres in planar (one grain thick samples) and fully 3D DEM samples. In Step 2, we summarize the properties of the sample: network connectivity and individual bond properties (i.e., fracture strength, fracture surface energy, size, realized tensile force and strain energy). This summary is compiled into an input data to Step 3, where a data-driven and multiscale network flow analysis is undertaken to predict the ultimate macrocrack path ${\mathcal {C}}$ based on a prescribed fracture criteria across the different loading stages from pre-peak to the post-peak softening regime. Eight fracture criteria are examined with respect to their ability to deliver an early prediction of ${\mathcal {C}}$ in the prefailure regime. Criteria 1 and 2 correspond to the bottleneck of the strain energy flow network and its residual, respectively; while Criteria 3 and 4 focus on the bottleneck of the tensile force flow network and its residual. We recast into a network flow formulation four widely used fracture criteria: minimum energy functional of Francfort and Marigo^31^, minimum critical strain energy density^15^, maximum energy release rate^32^, maximum normal stress (Rankine stress)^33^. In Step 4, assuming specific conditions hold, we present closed-form relationships between the eight criteria which, in conjunction with the numerical predictions from Step 3, characterize explicitly the influences of disorder in the network connectivity, bond heterogeneities in fracture strength and surface energy, and stage of loading on ${\mathcal {C}}$. Our hypothesis is that since force and energy are correlated and share common transmission paths, their flow bottleneck may be the precursory failure mechanism that can reconcile and consolidate these optimality criteria in one unified framework.

## Theory

### Construction of the flow networks

At each stage of loading $\epsilon$, we construct three *flow networks* $\mathcal {F}, \mathcal {F}_1$ and $\bar{\mathcal {F}}$ from the bond contact network ${\mathcal {N}}$. The flow network $\mathcal {F}=(G,s,t,c)$ is given in terms of: $G=(V,A)$ which is a directed graph that consists of a set of nodes $v \in V$ and a set of arcs $e \in A$; a source node *s*; a sink node *t*; and a *capacity function* $c:A\rightarrow \mathbb {R}_0^+$. A *flow* is a function that assigns non-negative real numbers to the arcs of *G*, $\phi :A\rightarrow \mathbb {R}_0^+$, subject to the following conditions.

**Figure 3:**
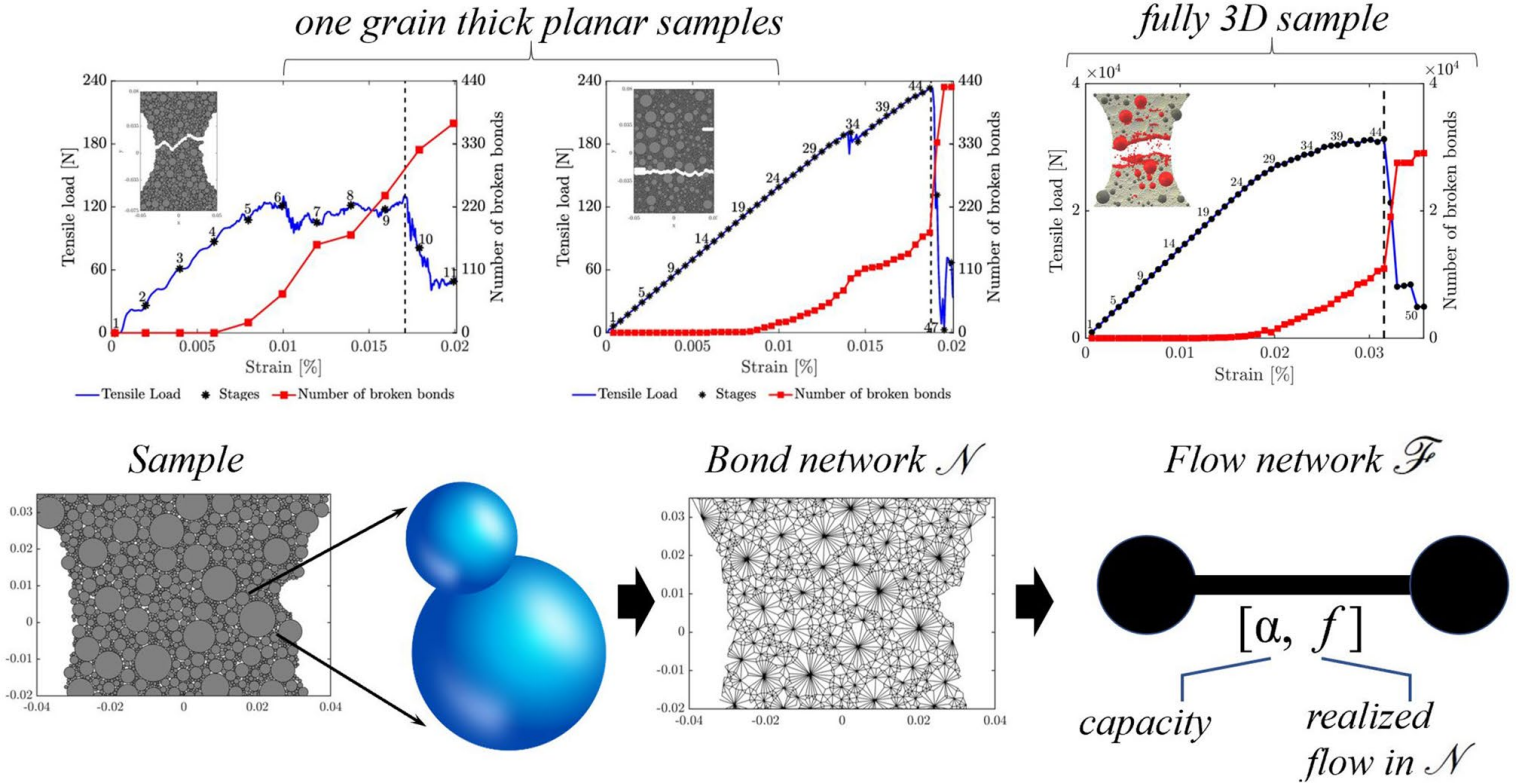
(Color online) Microstructural data were derived from DEM simulations of the experiments of van Vliet and van Mier^34^ on concrete under uniaxial tension, with attention paid to the mesoscale structure of real concrete. Data at each stage of loading are mapped to flow networks to study energy and force transmission across scales.

- **Capacity constraint: **for every arc, $e\in A$, the flow cannot exceed the capacity
  $$0\le \phi (e)\le c(e).$$
- **Conservation of flow: **for every node except the source and sink, $v \in V - \{s,t\}$, the amount of flow entering a node is the same as that leaving the node viz.
  $$\displaystyle \sum _{e\in \delta ^+(v)}\phi (e)=\sum _{e\in \delta ^-(v)}\phi (e)$$
  where $\delta ^-(v)$ is the set of arcs terminating at *v*, and $\delta ^+(v)$ is the set of arcs emanating from *v*.

Next we relate $\mathcal {F}$ to the bond contact network ${\mathcal {N}}$ by assigning the nodes *v* to represent the grains while the arcs *e* represent the bonds (Fig. 3). Each bond in ${\mathcal {N}}$ has two properties: a bond flow *f* which changes with the stage of loading and a fixed capacity $\alpha$. Both *f* and $\alpha$ are known from the DEM data and form part of the input to the network flow analysis. We consider two types of flow: (i) energy flow, (ii) tensile force flow. These flows can be reasonably assumed to obey the above two conditions for flow^16^.

For energy transmission, the bond flow corresponds to the potential energy in the bond *U*: $f(e,\epsilon )=f_E(e,\epsilon )=U(e,\epsilon )$. The bond capacity is given by the Griffith’s fracture surface energy $\gamma$^14^:

1 $$\begin{aligned} \displaystyle {\alpha (e)=\alpha _E(e)=\gamma (e)=\frac{(F^n_{min})^2}{2K_n} = \frac{ (r_A+r_{B}) (F^n_{min})^2}{4 E {r_A}{r_B}}}, \end{aligned}$$

where $K_n = E \frac{2r_A r_B}{r_A + r_B}$ is the normal contact stiffness and $F^n_{min}$ is the bond strength, the force needed to break the bond, given by

2 $$\begin{aligned} \displaystyle {F^n_{min}(e)=\frac{T_n(e) b(e)}{ \pi }}, \end{aligned}$$

*E* is the Young’s elastic modulus of the contact, $T_n$ is the critical normal tensile stress for the contact, *b* is the bond contact area $b=\pi r_{A}^2$, and $r_A$ and $r_{B}$ are the radii of the grains sharing the bond such that $r_{A} \le r_B$ (for convenience, we refer to $r_{A}$ simply as *r* in the rest of the paper). For force transmission, the bond flow is the tensile force magnitude $f(e,\epsilon )=f_F(e,\epsilon )=||\mathbf {F}_n(e)||$, while the capacity is given by the bond strength $\alpha (e)=\alpha _F(e)=F^n_{min}(e)$. All the material properties have been calibrated against the physical experiments of van Vliet and van Mier^34^ (see Nitka and Tejchman^35^ for complete details). In all the samples, we assume all bonds are in tension and no new contacts are created, following Tordesillas et al.^16^. Therefore $A(m) \subseteq A(m-1) \subseteq \ldots . \subseteq A(1)$, where *m* is the final stage of loading.

A feasible crack path (cut) of $\mathcal {F}$ can be defined as two distinct partitions $\{W,W'\}$ of *V* such that $s\in W$ and $t\in W'$. Every cut of $\mathcal {F}$ determines a set of links $\Gamma$ of *G*, where $\Gamma$ contains all arcs emanating from a node in *W* and terminating on a node in $W'$. The capacity of such a cut $\Gamma$ is defined as $c(\Gamma ) = \displaystyle \sum _{e\in \Gamma }{\alpha (e)}.$ Here we are interested in finding cuts $\Gamma$ of $\mathcal {F}$ which satisfy specific optimality conditions on their capacity $c(\Gamma )$. We focus on the following two types of cuts of $\mathcal {F}=(G,s,t,c)$:

- The Minimum Cut of $\mathcal {F}=(G,s,t,c)$ is the cut $\Gamma _{min}$ such that
  $$c(\Gamma ) = Minimize\big \{ \displaystyle \sum _{e\in \Gamma }{\alpha (e)} \big \}.$$
- This cut corresponds to the *bottleneck* of the flow network, $B(\mathcal {F}(G,s,t,c))=\Gamma _{min}$. This is obtained by solving the Max-flow Min-cut Problem using the Ford-Fulkerson algorithm^36^.
- The Maximum Cut of $\mathcal {F}=(G,s,t,c)$ is that cut $\Gamma _{max}$ such that
  $$c(\Gamma ) = Maximize\big \{ \displaystyle \sum _{e\in \Gamma }{\alpha (e)} \big \}.$$
  The problem of finding an optimal solution is NP-hard, so we use the probabilistic heuristic optimization algorithm of simulated annealing to solve this approximately for $\mathcal {F}$^37^.

In addition to $\mathcal {F}$, we define two additional flow networks $\mathcal {F}_1$ and $\bar{\mathcal {F}}$ with the same topology as $\mathcal {F}$ but which differ in their capacities as follows: unit capacity flow network $\mathcal {F}_1 = (G,s,t,1)$ and the residual flow network $\bar{\mathcal {F}} = (G,s,t,\bar{\alpha })$ where $\bar{\alpha }= \alpha -f.$

### Criterion 1: The energy flow bottleneck as the path of minimum Griffith’s fracture surface energy^14^

Griffith postulated that a crack will propagate when there is sufficient stored potential energy to overcome the fracture surface energy of the material. The outstanding question is: *Once this condition is met, along which path will the ultimate macrocrack propagate? * Here we propose that this path is given by $B(\mathcal {F}_E)$, the bottleneck of the flow network $\mathcal {F}_E = (G,s,t,\gamma )$: the cut where the Griffith’s fracture surface energy $\gamma (\Gamma )$ is minimum over all feasible cuts $\Gamma$ of $\mathcal {F}_E$.

### Criterion 2: The residual energy flow bottleneck

Criterion 1 can be extended to account for the state of flow in the system at $\epsilon$, by incorporating the realized strain energy for the bond $U(\epsilon )$ in the capacity function: $\bar{\alpha }_{E}(\epsilon )= \gamma - U(\epsilon )$. Thus an alternative criterion for the crack path is that path which is closest to breaking point relative to the fracture surface energy of the system: the bottleneck $B(\bar{\mathcal {F}}_E)$ of the flow network $\bar{\mathcal {F}}_E = (G,s,t,\gamma - U)$.

### Criterion 3: The force flow bottleneck as the path of minimum fracture strength

The path of minimum fracture strength is given by the force bottleneck $B(\mathcal {F}_F)$: the cut along which the sum of member bond strengths is minimum over all feasible cuts of $\mathcal {F}_F = (G,s,t,F^n_{min})$.

### Criterion 4: The residual force flow bottleneck

The path that is closest to breaking point relative to the fracture strength of the system can be established from the bottleneck $B(\bar{\mathcal {F}}_F)$, where $\bar{\mathcal {F}_F}=(G,s,t,F^n_{min}-f_F)$. The same arguments from Criterion 2 applies. Since the force flow is only approximately conserved in ${\mathcal {N}}$, $B({\mathcal {F}}_F) \approx B(\bar{\mathcal {F}}_F)$.

### Criterion 5: The path of minimum Francfort–Marigo energy functional^31^

Francfort and Marigo^31^ proposed that the macrocrack develops along the path where the total energy functional $E_{tot}$ is minimum, where

3 $$\begin{aligned} E_{tot}(\Gamma , \epsilon ) = E_{strain}({\mathcal {N}} \setminus \Gamma , \epsilon ) + \gamma (\Gamma ), \end{aligned}$$

$E_{strain}({\mathcal {N}} \setminus \Gamma , \epsilon )$ is the sum of elastic strain energies stored in all the bonds in ${\mathcal {N}}$ excluding those in the virtual cut path $\Gamma$, and $\gamma (\Gamma )$ is Griffith’s fracture surface energy (or energy dissipated due to crack formation) of the cut $\Gamma$. Equation 3 can be expressed as

4 $$\begin{aligned} E_{tot}(\Gamma )=\sum _{e\in A \backslash \Gamma }U(e)+\sum _{e\in \Gamma }\gamma (e) = \sum _{e\in A} U(e)+\sum _{e\in \Gamma } \big (\gamma (e)-U(e) \big ). \end{aligned}$$

Since $\displaystyle \sum _{e\in A}U(e)$ does not depend on $\Gamma$, $E_{tot}$ is minimized when $\displaystyle \sum _{e\in \Gamma } \big (\gamma (e)-U(e) \big )$ is minimized. This path is given by the the bottleneck $B(\bar{\mathcal {F}}_E)$ of the flow network $\bar{\mathcal {F}}_E = (G,s,t,\gamma - U)$ from Criterion 2.

### Criterion 6: The path of minimum critical strain energy density

Building on Criterion 1, the capacity function given in Equation 1 can instead be expressed in terms of the critical strain energy density in the spirit of Sih^15^: $\alpha _E(e)= \hat{\gamma }(e) =\frac{\gamma (e)}{\pi (r(e))^2}$. This yields a new potential crack path which is given by the minimum cut of the flow network $\mathcal {F}=(G,s,t,\hat{\gamma })$. Thus, the path where the Griffith’s fracture surface energy density $\hat{\gamma }(\Gamma )$ is minimum is given by the minimum cut of $\mathcal {F}(G,s,t,\frac{\gamma }{\pi r^2 })$, $B\big ( \mathcal {F}(G,s,t,\frac{\gamma }{\pi r^2} )\big )$.

### Criterion 7: The path of maximum energy release rate^32^

To find the path along which the maximum potential energy is released in ${\mathcal {N}}$, we solve for the maximum cut $\Gamma _{max}$ of $\mathcal {F}_E$ such that

$$c\big (\Gamma _{max}\big )= Maximize\displaystyle \Big \{ \sum _{e\in S(\Gamma )}\gamma (e) \Big \};$$

here $S(\Gamma (\epsilon ))$ is the set of saturated edges of $\Gamma (\epsilon )$. That is, $S(\Gamma (\epsilon ))$ comprises those broken bonds in $\Gamma (\epsilon )$ as a result of the bond flow at $\epsilon -1$ reaching its respective Griffith’s fracture surface energy, $f_E(\epsilon -1)=\gamma (\epsilon -1)$.

### Criterion 8: The path maximum normal stress (Rankine stress criterion^33^)

To find the path that satisfies the Rankine stress criterion in ${\mathcal {N}}$, we solve for the maximum cut $\Gamma _{max}$ of $\mathcal {F}_F$ such that

$$c\big ( \Gamma _{max} \big )= Maximize\displaystyle \Big \{ \sum _{e\in \Gamma }{ \frac{ ||\mathbf {F}_n(e)|| }{\pi (r(e))^2 }} \Big \}.$$

### Corollaries of conservation of energy and force flow in the bond network

The actual strain energies and tensile force magnitudes realized in the DEM simulations are only approximately conserved in ${\mathcal {N}}$. Here we present Step 4 (recall Fig. 1), where we assume that conservation of flow is exactly satisfied which implies that: (i) the total flow across any two feasible cuts of a flow network is the same; (ii) the bottleneck of any two flow networks with capacities that differ only by a multiplicative constant is the same. Below, subject to these two conditions, we establish closed-form relationships between the energy flow bottleneck $B(\mathcal {F}_E)$ from Criterion 1, the force flow bottleneck $B(\mathcal {F}_F)$ from Criterion 3, and the paths corresponding to all the other fracture criteria. Note that corollaries A–E apply to all stages of loading history, whereas corollary F is confined to the post-peak softening regime.

#### A. The path of least resistance to fracture is the path closest to fracture, with respect to either the fracture strength or the fracture surface energy of the system: $B(\bar{\mathcal {F}}_E) = B(\mathcal {F}_E)$ and $B(\bar{\mathcal {F}}_F) = B(\mathcal {F}_F)$.

To prove this, recall that by the definition of the minimum cut, we have

$$c\big (B(\bar{\mathcal {F}}_E)\big )= Minimize\big ( \displaystyle \sum _{e\in \Gamma }(\gamma (e)-U(e))\big ) = Minimize\big ( \displaystyle \sum _{e\in \Gamma }\gamma (e)- \displaystyle \sum _{e\in \Gamma }U(e)\big ).$$

Since flow is conserved, the energy flow for any feasible path $\Gamma$ in $\mathcal {F}_E$ is the same: $\displaystyle \sum _{e\in \Gamma }U(e)$ is the same for any $\Gamma$. Therefore $\displaystyle \sum _{e\in \Gamma }\gamma (e)- \displaystyle \sum _{e\in \Gamma }U(e)$ is minimized when $\displaystyle \sum _{e\in \Gamma }\gamma (e)$ is minimized. But $\displaystyle \sum _{e\in \Gamma }\gamma (e)$ is minimized at $B(\mathcal {F}_E)$. Thus $B(\bar{\mathcal {F}}_E) = B(\mathcal {F}_E)$. By the same argument, $B(\bar{\mathcal {F}}_F) = B(\mathcal {F}_F)$.

#### B. The path with the minimum energy functional as defined by Francfort and Marigo^30^ is the path with the minimum fracture surface energy.

$E_{tot}$ is minimized along the bottleneck of the residual network $\bar{\mathcal {F}}_E = (G,s,t,\gamma - U)$ from Criterion 2 which, as shown in Corollary A, is given by $B(\mathcal {F}_E)$ from Criterion 1.

#### C. If the bonds are uniformly sized, then the path with the minimum critical strain energy density is the path with the minimum fracture surface energy.

If the bonds are uniformly sized, then the two flow networks $\mathcal {F}(G,s,t,\frac{\gamma }{\pi r^2})$ and $\mathcal {F}(G,s,t,\gamma )$ will have the same bottleneck as their capacity functions differ only by a constant multiple. That is, the critical strain energy density is minimized along $B\big ( \mathcal {F}_E \big )$ from Criterion 1.

#### D. When the bonds have the same critical tensile bond stress, then the path that satisfies the Rankine stress criterion is the path with the minimum fracture strength.

Observe that, regardless of stage of loading, the path which maximizes the tensile normal stress in $\mathcal {F}_F(G,s,t,F_{min}^n)$ is that path with the least total bond area since all cut flows in the flow network are the same. This path is given by $B\big (\mathcal {F}_F(G,s,t,b)\big )$, the minimum cut of the flow network $\mathcal {F}_F(G,s,t,b)$ with edge capacity equal to the area of the corresponding bond *b*. Since $F_{min}^n(e)=\frac{T_n (e)}{\pi } b(e)$, in general, $B\big (\mathcal {F}_F(G,s,t,F_{min}^n)\big ) \ne B\big (\mathcal {F}_F(G,s,t,b)\big )$. But if the critical tensile normal contact stress $T_n$ is the same for every bond in ${\mathcal {N}}$, then the capacity functions will be different only by a multiplicative constant. Thus $B\big (\mathcal {F}_F(G,s,t,F_{min}^n)\big )$ = $B\big (\mathcal {F}_F(G,s,t,b)\big )$. That is, when the critical tensile stress $T_n$ is uniform in ${\mathcal {N}}$, the path that satisfies the Rankine stress criterion is the force bottleneck $B\big (\mathcal {F}_F\big )$ from Criterion 3.

#### E. When bond properties are uniform, and the only source of heterogeneity is the bond connectivity—viz. disordered but otherwise homogeneous network—then all the fracture criteria except for the maximum release rate (Criterion 7) are satisfied along that path with the least number of bonds.

In the special case when bonds in ${\mathcal {N}}$ are homogeneous, the capacity functions $\alpha _E$ and $\alpha _F$ are both constants and $B(\mathcal {F}_E) = B(\mathcal {F}_F)$. Moreover, the paths from Criteria 1–6 and 8 converge to one path, which is given by the minimum cut of $\mathcal {F}_1(G,s,t,1)$, the flow network with unit capacity. This path, the so-called minimum edge cut, is the cut with the least number of bonds^38^. The number of bonds in this cut, $p_{min}$, gives a measure of path redundancy in ${\mathcal {N}}$: the minimum number of all available flow pathways (joint and disjoint) between the top and bottom walls of the specimen.

#### F. In the post-peak softening regime, when the maximum flow is reached, then the path of maximum energy release rate is the same as the path with the minimum fracture surface energy.

**Figure 4:**
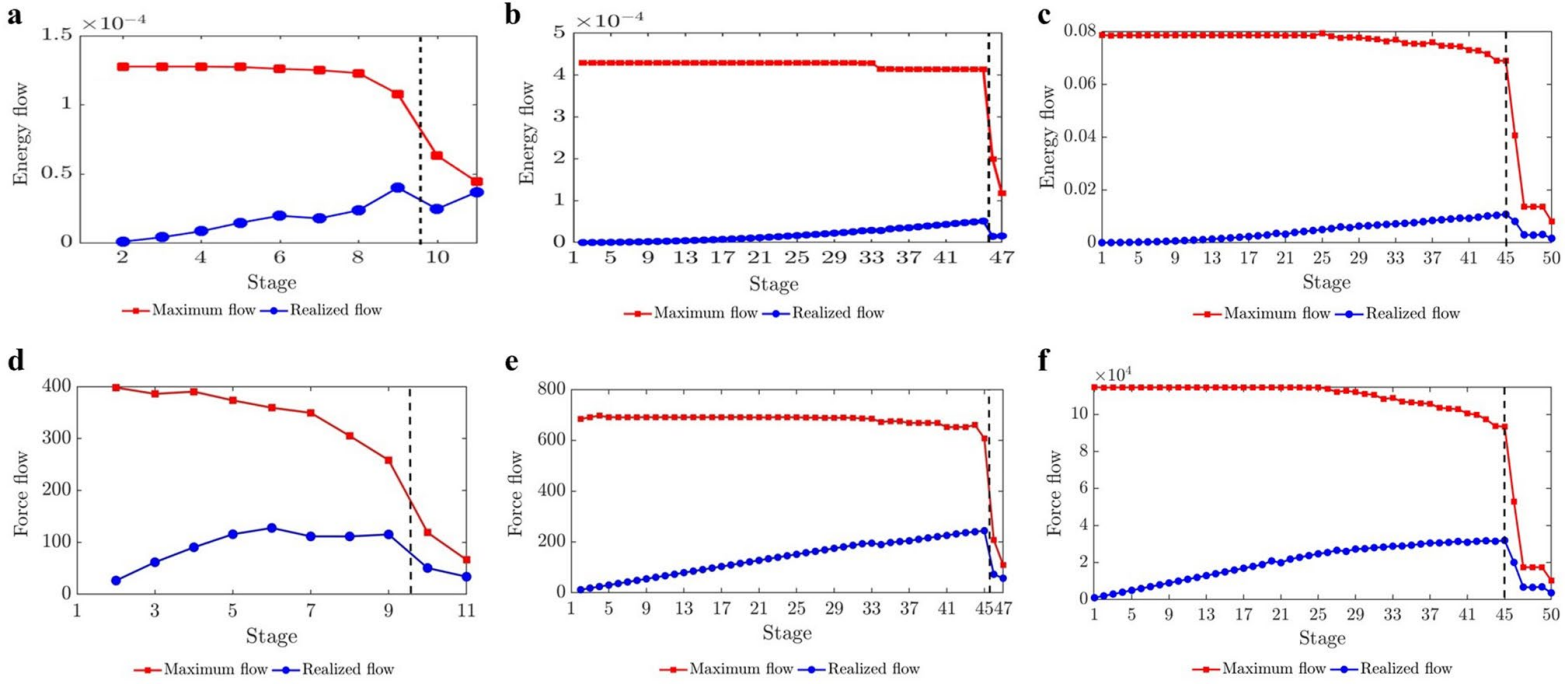
(Color online) *Failure is characterized by a transmission of energy and force near the global flow capacity of the bond network*. (**a**,**d**) D1. (**b**,**e**) D2. (**c**,**f**) D3. Evolution of the realized flow and the maximum flow $\Phi _{max}(\epsilon )$ in ${\mathcal {N}}$: energy flow (**a**,**b**,**c**), force flow (**d**,**e**,**f**). Dashed vertical line marks the stage at peak load. Note that the sample is only partially split and some bonds remain in the macrocrack path at the final stage *m*.

From Fig. 4, we see that the post-peak softening regime is distinguished by the realized flow being close to the maximum flow $\Phi _{max}$ in $\mathcal {F}_E$. The maximum flow is a flow $\phi$ such that the net flow leaving the source node $\Phi (\{s\})$ is maximized. Let $\Theta (\Gamma (\epsilon ))$ be defined as the total flow from the saturated edges in $\Gamma$: $\Theta (\Gamma )=\displaystyle \sum _{e\in S(\Gamma )}\phi (e)=\displaystyle \sum _{e\in S(\Gamma )}\gamma (e)$. Since $\Phi (\Gamma )$ is the total flow across $\Gamma$, $\Theta (\Gamma )\le \Phi (\Gamma )$. By flow conservation, the total flow through any feasible cut is the same, $\Phi (\Gamma )=\Phi (\Gamma _{min})$. Since $\Gamma _{min}$ is a minimum cut, every edge of $\Gamma _{min}$ is saturated when the maximum flow is reached in ${\mathcal {N}}$. That is, $S(\Gamma _{min})= \Gamma _{min}$ and $\Theta (\Gamma _{min})=\displaystyle \sum _{e\in \Gamma _{min}}\phi (e)=\displaystyle \sum _{e\in \Gamma _{min}}\gamma (e)=\Phi (\Gamma _{min})$. Thus $\Theta (\Gamma )\le \Theta (\Gamma _{min})$. Given $\Gamma$ is arbitrary, this implies that at those stages where the realized flow in ${\mathcal {N}}$ reaches the maximum flow $\Phi _{max}$, the energy release is maximized along $B(\mathcal {F}_E)$ from Criterion 1.

## Results and discussion

**Figure 5:**
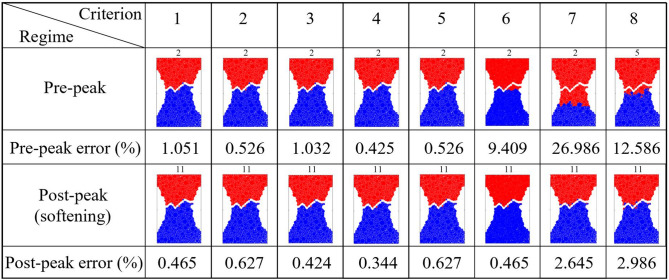
(Color online) *Early prediction of the final crack path* ${\mathcal {C}}$ *by the energy, force and residual bottlenecks.* Results from Step 3 for sample D1. Summary of crack path predictions (red-blue interface) for the eight fracture criteria and their maximum error in the pre-peak and post-peak softening regimes (to aid visual, an artificial separation shows ${\mathcal {C}}$). Representative stages shown are stage 2 when the system spanning force chain network is first established and the final stage of loading. Criterion 8 starts at stage 5 as there are no broken bonds until then.

**Figure 6:**
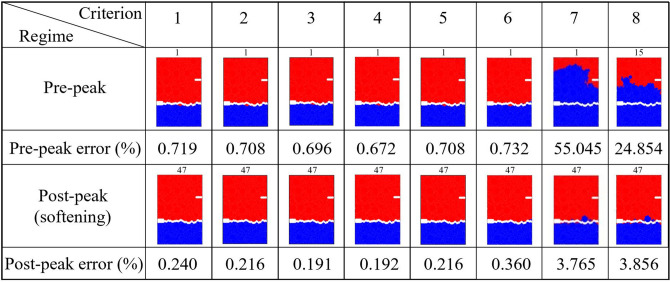
(Color online) *Early prediction of the final crack path* ${\mathcal {C}}$ *by the energy, force and residual bottlenecks.* Results from Step 3 for sample D2. Summary of crack path predictions (red-blue interface) for the eight fracture criteria and their maximum error in the pre-peak and post-peak softening regimes (to aid visual, an artificial separation shows ${\mathcal {C}}$). Representative stages shown are stage 1 when the system spanning force chain network is first established and the final stage of loading. Criterion 8 starts at stage 15 as there are no broken bonds until then.

**Figure 7:**
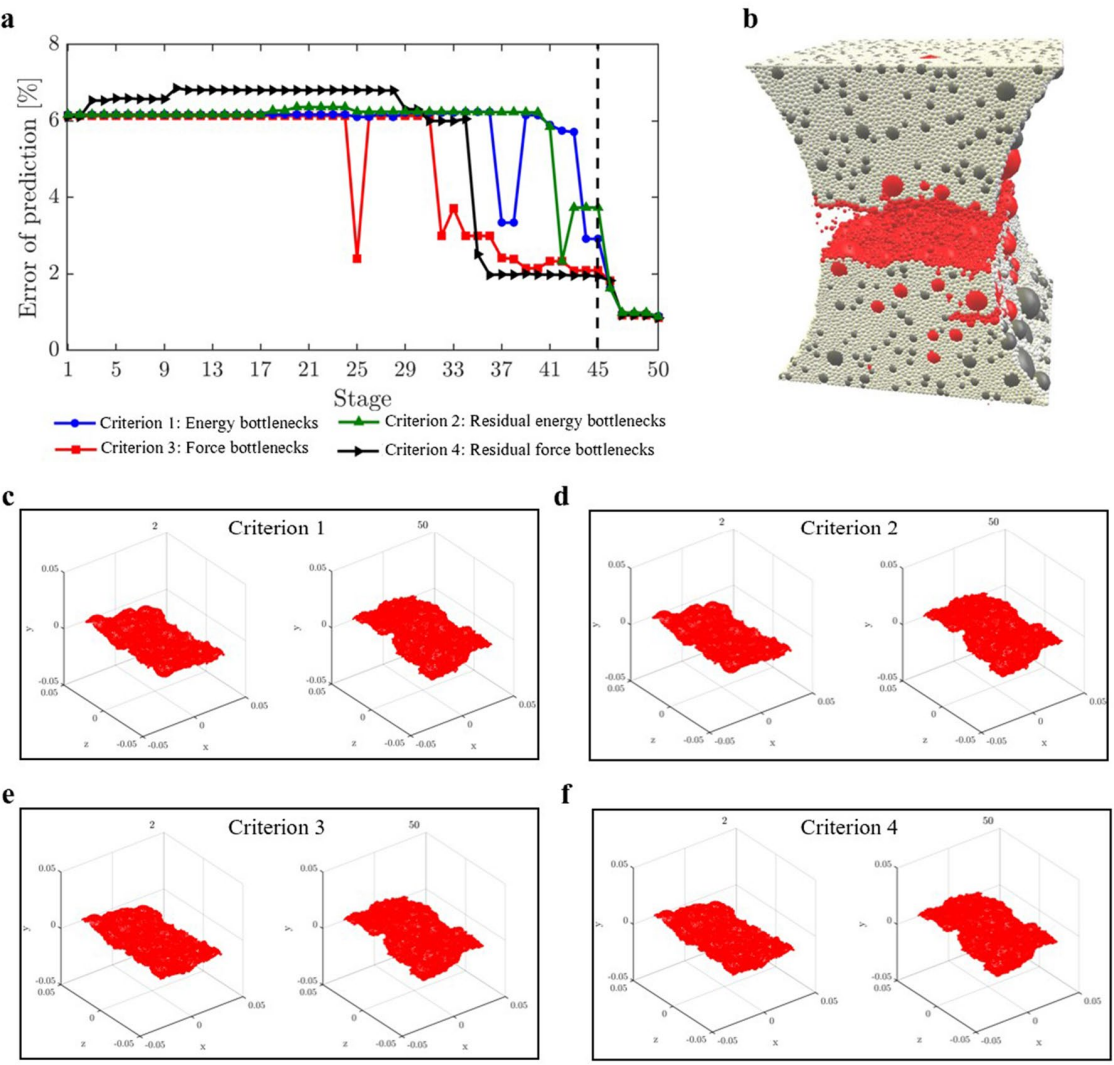
(Color online) *Early prediction of the final fracture surface* ${\mathcal {C}}$ *by the energy, force and residual bottlenecks.* Results from Step 3 for the 3D sample D3: (**a**) Evolution of the error of the prediction. (**b**) 3D-view of the sample with broken contacts (grains with broken contacts are colored red). Location of failure (here artificial separation is introduced to aid visual effects). (**c**–**f**) 3D surface view of the bottleneck at stage 2 and the final stage 50.

We sought to address an open question: Can the path of the crack that leads to failure be predicted from the connectivity of transmission paths and their microstructural fracture properties and, if so, how far in advance? That the energy and force bottleneck distinguish themselves from other feasible partitions of the specimen—even before the onset of damage—suggests this is possible (Figs. 5, 6 and 7). For each criterion, we quantified the error between the predicted location and the actual macrocrack path ${\mathcal {C}}$ as follows:

5 $$\begin{aligned} P = \frac{|(V_{B_u} \setminus V_{{{\mathcal {C}}}_u}) \cup (V_{{{\mathcal {C}}}_u}\setminus V_{B_u})|}{|V_T|} \times 100 \%, \end{aligned}$$

where $V_{B_u}$, $V_{{{\mathcal {C}}}_u}$ are the set of grains in the upper part of the bottleneck and the macrocrack respectively, $V_T$ is the set of all the grains in the sample, and $|\;\;|$ denotes the cardinality (number of grains). Hence $P$ quantifies the number of offset grains in the prediction relative to the macrocrack, normalized by the total number of grains.

**Figure 8:**
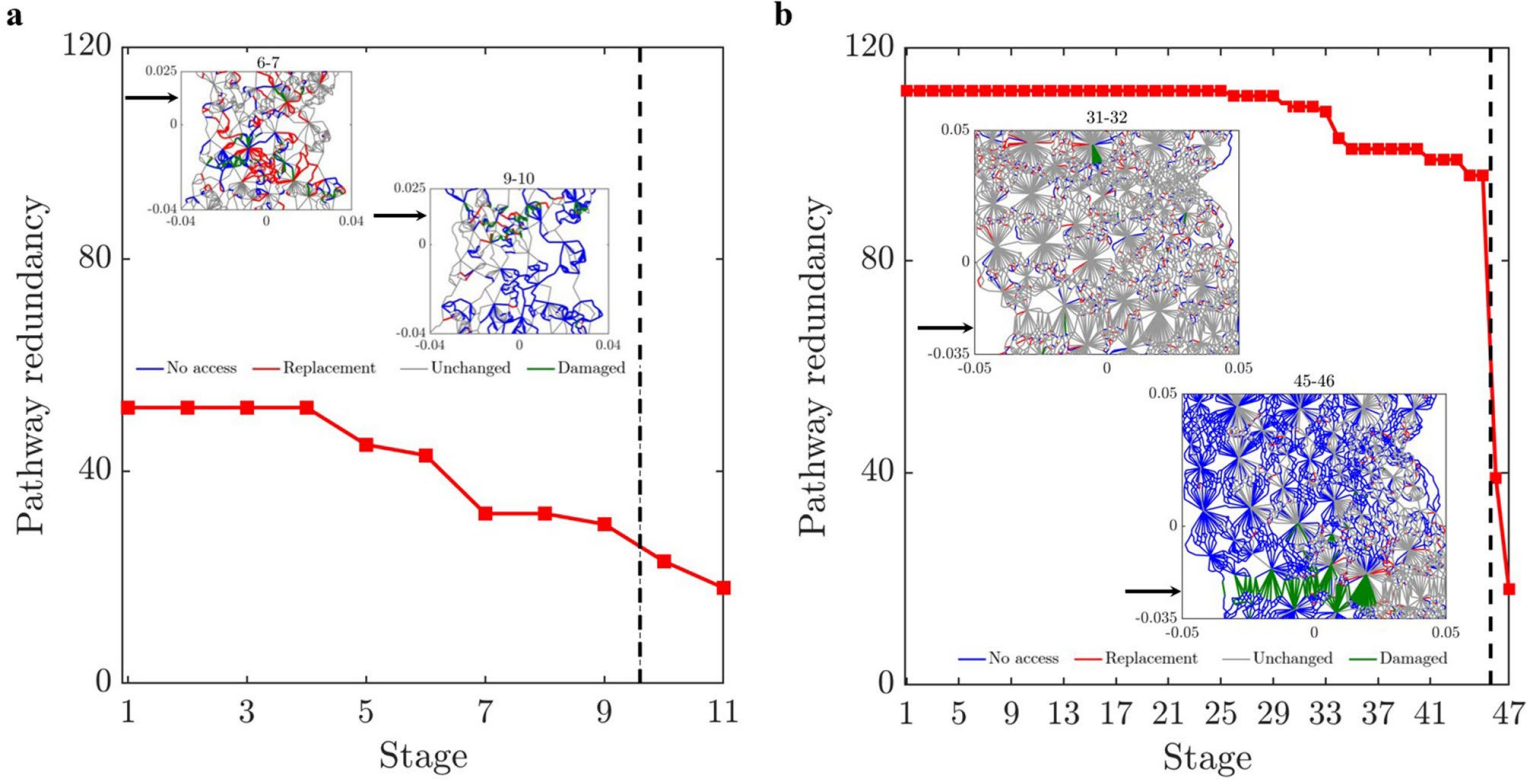
(Color online) *Prefailure dynamics is governed by pathway redundancy.* Evolution of the pathway redundancy $p_{min}$ for (**a**) D1 and (**b**) D2. Inset shows spatial distribution of links that leave $\mathcal {P}$ but remain in ${\mathcal {N}}$ (no access), enter $\mathcal {P}$ (replacement), unchanged and damaged links in $\mathcal {P}$ due to rerouting. Black arrow marks the general location of ${\mathcal {C}}$. Dashed vertical line marks the stage at peak load.

**Figure 9:**
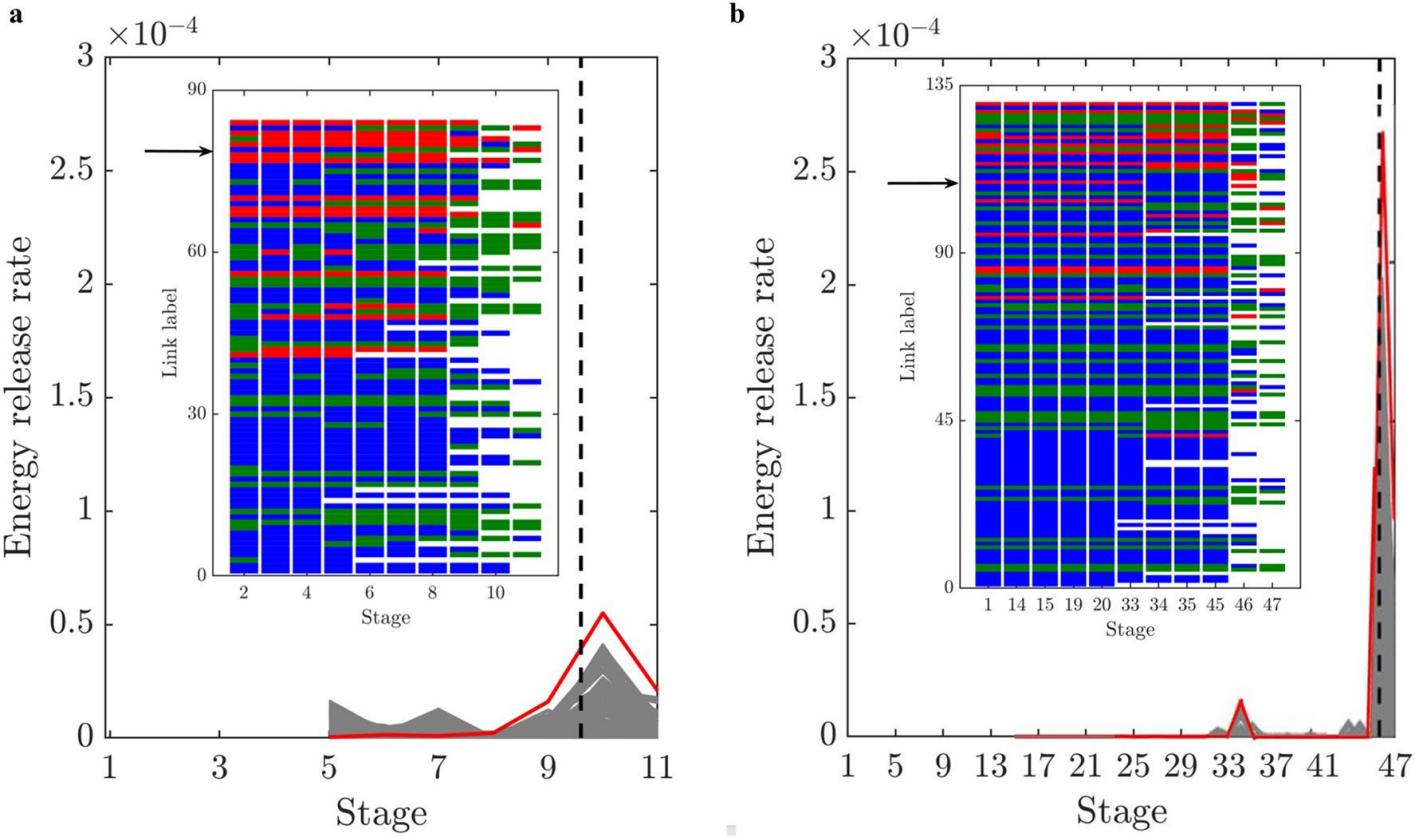
Evolution of energy release rate for all 10,000 random cuts (grey) and bottlenecks (red) for (**a**) D1 and (**b**) D2. No broken bonds until stage 5 for D1 and stage 15 for D2. Insets show the evolution of link type for each of the *k* member links of the energy bottleneck $B(\mathcal {F}_E)$. For D1, $k=84$; for D2, $k= 130$. Links in *B* are ranked from lowest to highest fracture surface energy (labeled 1 to *k*) at that stage when the tensile force chain network is first established (stage 2 for D1 and stage 1 for D2). Link type is represented by a horizontal bar colored according to the type of grains in contact: red (TT—tensile force chain grains), green (NN—neither is a tensile force chain grain), blue (TN—one is a tensile force chain grain, the other is not). Bonds having above the global mean fracture surface energy lie above the black arrow. A transition to a different link type manifests as a change in the color of the bar. No bar is shown for a link that breaks.

**Figure 10:**
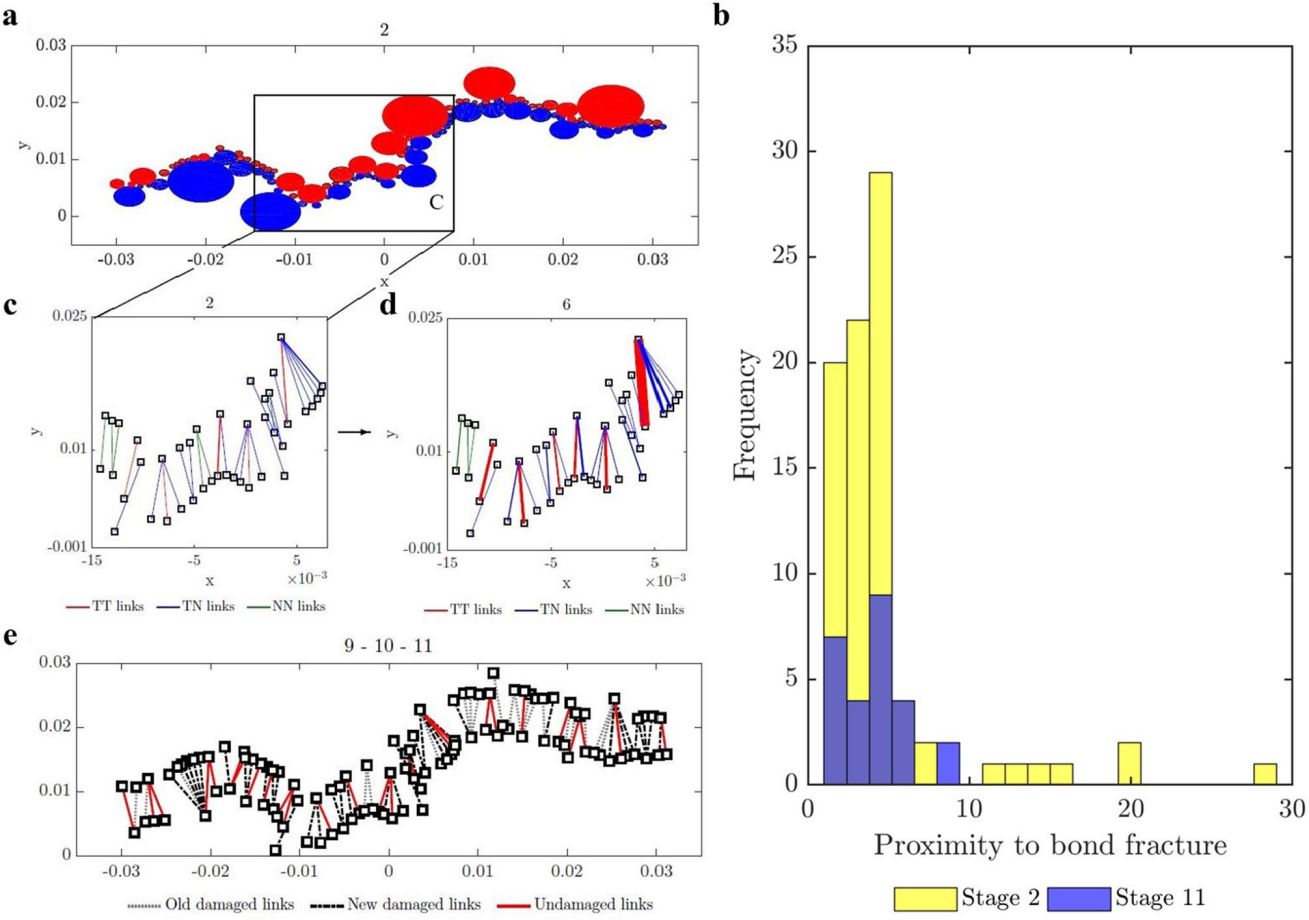
(Color online) *Heightened interdependency among bonds in* $B(\mathcal {F}_E)$ *predispose them to cascading failure*. (**a**) Grains colored red (blue) belong to the upper (lower) portion of the specimen in D1. (**b**) Proximity to fracture of bonds in $B(\mathcal {F}_E)$. Link types are colored red (TT—tensile force chain grains), green (NN—neither is a tensile force chain grain), blue (TN—one is a tensile force chain grain, the other is not) for the highlighted region in (**a**) at: (**c**) stage 2 and (**d**) stage 6. Recall Stage 2 is when the tensile force chain network is first established and stages 2–6 see a steady increase in the applied tensile load. Line thickness is proportional to the magnitude of the contact force. (**e**) Evolution of bonds as failure cascades in $B(\mathcal {F}_E)$ across stages 9–11 in D1. Similar trends apply to D2 (not shown).

1. We showed that the bottlenecks of the energy and force flow network and those from their respective residual networks are essentially the same. This implies that at any given stage of loading, the bottlenecks distinguish themselves from all other feasible crack paths in two respects: by having the least total capacity (Griffith’s fracture surface energy in $\mathcal {F}_E$, or fracture strength in $\mathcal {F}_F$), and by being closest to this capacity. That is, the fracture criterion that delivers an early prediction of ${\mathcal {C}}$ must be related to the flow bottleneck, the path of least resistance to fracture, where the force and energy flows are also closest to their critical fracture values. On the other hand, the realized (actual) flows are an outcome of an optimized transmission process that maximizes the global throughput subject to the redundancies of, and the capacities of member segments or bonds along, the percolating paths through the specimen. Results here are consistent with recent experiments on ceramics^3, 4^, metallic glasses^6^ and graphite^39^.
2. The best of the eight fracture criteria is the force residual bottleneck (Criterion 4). This makes sense because: (i) it uses the actual bond capacities from the DEM model; and (ii) it accounts for the current state of the material as given by the actual flow realized at the given stage of loading. Since the bottleneck for Criterion 5 is found by solving the same minimum cut in Criterion 2, their errors are the same. That the error in D3, which initially has 1,396,172 links (c.f. 12,350 links in D1), is generally higher compared to the planar samples is likely due to roundoff error in the link capacities. Specifically, solving the Max-flow Min-cut Problem using the Ford-Fulkerson algorithm requires integer link capacities^40^, hence we round down the capacities to the nearest integer. In applications, the input data from Steps 1-2 would ultimately dictate which bottleneck can be computed and used for early prediction of ${\mathcal {C}}$ in Step 3 (recall Fig. 1).
3. The paths of minimum critical strain energy density, maximum energy release rate and maximum normal stress (Criteria 6–8) do not provide a reliable early prediction of the failure location. This is due to path redundancy in ${\mathcal {N}}$ in the prefailure regime, which is not captured by any of these fracture criteria. Redundancies in transmission paths are what enables energy and force to be rerouted to higher capacity bonds to prevent bond breakage. As shown recently^16^, damage in the early stages of of the prefailure regime mainly occurs away from the macrocrack path ${\mathcal {C}}$. Similar stress redistributions around strain concentration sites have been observed to divert damage away from the crack path (viz., the crack tip) into the bulk of the sample^9, 41^. Given that ${\mathcal {C}}$ essentially coincides with the energy bottleneck $B(\mathcal {F}_E)$, this may initially seem counterintuitive given the close proximity to fracture of the bonds in $B(\mathcal {F}_E)$. It turns out there is an interplay between force chain stability in the energy bottleneck and path redundancy, as we now demonstrate.
  We first quantify path redundancy through $p_{min}$^36^, the minimum number of available transmission pathways between the top and bottom walls of the specimen (Fig. 8). $p_{min}$ is purely a topological property of ${\mathcal {N}}$ and is equal to the number of percolating link-disjoint paths (paths that have no common links) through ${\mathcal {N}}$ between the source and the sink. Broadly, $p_{min}$ is one of the fundamental concepts in measuring resilience in transmission networks, viz. the ability of a network to withstand ‘attacks’ (loss of links or nodes). For instance, in road networks, $p_{min}$ may be used to quantify the extent to which cars can be rerouted to alternative paths when a road is closed to traffic (e.g., roadworks or accident). The monotonic decrease in $p_{min}$ reflects the degradation of flow paths caused by the spread of damage (Fig. 3). Damage disrupts the transmission of energy and force through the specimen since the breakage of a bond disconnects percolating paths for flow. In the presence of redundant paths, this disruption triggers a reroute or redistribution of flow. We highlight this process in Fig. 8-inset in terms of the optimized force route $\mathcal {P}$, the most direct or shortest possible percolating routes for force transmission, where most force chains develop^16^. As seen in Fig. 8-inset, the system recovers in the presence of damage. New links in $\mathcal {P}$ replace old links therein which either break or can no longer be accessed. When $p_{min}$ is relatively high, just after the onset of damage in ${\mathcal {N}}$ (stages 6–7 for D1; stages 31–32 for D2), the system compensates by replacing contacts that can no longer be accessed in $\mathcal {P}$ (numbers of red and blue contacts are roughly balanced). By contrast, in the transition to the post-peak softening regime (stages 9–10 for D1; stages 45–46 for D2), $\mathcal {P}$ rapidly degrades without a matching recovery: observe the surge in population of blue contacts as that of feasible replacement contacts in red dwindle.
  Remarkably, while the bottleneck force chains are the most obvious suspect locales for incipient failure, we find these endure during the prefailure regime, contributing mainly to the accumulation of stored energy and consequent energy release in the terminal phase of rapid fracture (Fig. 9). Observe in Fig. 9-inset that prefailure damage is confined to low capacity links, suggesting that forces are spread out across member contacts (Fig. 10a–d). As previously demonstrated^16^, there is a process of cooperative evolution between the preferred paths for damage (i.e., bottleneck) and the preferred paths for force transmission (i.e., force chains) that underlies material robustness in the prefailure regime. Damage being confined to low capacity links in the bottleneck means that not only is the reduction in the global transmission capacity effectively minimized but also that high capacity contacts are left to support the remaining tensile force chains in the bottleneck. This coevolution among preferred paths for damage and force is an example of the so-called compromise-in competition between dominant mechanisms in the mesoregime of complex systems^42^. Evidence here suggests that this may be a contributing factor to the so-called ‘crack shielding’ or crack toughening that has been reported in the literature on granular composites^9,41,43^.
  The presence or lack of ITZs has no effect on the outcome of the analysis. When ITZs are present, the case with D2, we found that 60% of the contacts in the bottleneck are ITZ. This is consistent with prior studies which showed that ITZs tend to form attractors for macrocracks^27–30^. That is, they present paths of least resistance for crack growth, which evolve to ultimately span the sample through coalescence of ITZ microcracks (interconnected broken links along ITZ contacts)^24–30^.
4. While the antecedent dynamics described above protects the bottleneck from damage in the prefailure regime, it inevitably elicits the opposite effect by predisposing the bottleneck to rapid fracture in the softening regime. As path redundancy progressively drops with the spread of damage—the remaining bottleneck bonds are collectively brought closer and closer to their respective breaking points (Fig. 10b). A cascade of bond breakages then ensues, precipitating the abrupt transition to post-peak softening regime, with two attendant mechanisms *in the bottleneck* $B(\mathcal {F}_E)$:
  energy release rate becomes maximum (Fig. 9). This burst to a peak in the energy released in the transition to, and during, post-peak softening can be explained by the remaining high capacity bonds of tensile force chains and their lateral supports, since these store the highest levels of potential strain energy while being closest to fracture (Fig. 10). In contrast, concurrent energy release rates in all other feasible crack paths are much less, due to member bonds being further away from their respective fracture surface energies and hence are able to support an increase in strain energy without breaking. Note the higher energy released during failure cascade in D2 compared to that in D1, evident in Fig. 9, due to the higher pathway redundancy in D2 (Fig. 8). Tensile force chains in D2 are significantly more supported (higher connectivity means higher redundancy) than those in D1^16^, in turn enabling the D2 bottleneck to store higher strain energies in the stages preceding rapid fracture. This influence of connectivity on the stability of force chains was recently observed by Tang et al.^43^ in their study of packing and grain size distribution for optimal performance of reactive materials like aluminumpolytetrafluoroethylene (Al-PTFE) granular composites.
  normal stress becomes maximum as the tensile force flow through the bottleneck becomes close to the maximum force flow while the total bond area becomes minimum.
5. In the previous section, we derived closed-form relationships based on the result that force and energy bottlenecks provide an early and accurate prediction of the ultimate crack path ${\mathcal {C}}$ (step 4 in Fig. 1). These relationships naturally shed light on the specific influences of heterogeneities in bond geometry, strength and surface energy, topological disorder in the bond network, as well as the stage of loading on ${\mathcal {C}}$, as outlined in the previous section. Additionally, it can help explain why all eight fracture criteria approximately agree in their prediction of ${\mathcal {C}}$ in the post-peak softening regime. That is, the collective breakage of bonds along ${\mathcal {C}}$ in this regime results in ${\mathcal {C}}$ being identified: by criterion 8 due to corollary F, by criterion 7 due to corollary D combined with ${\mathcal {C}}$ becoming the path with the least total bond area, and by criterion 6 since ${\mathcal {C}}$ also becomes the path with the least the number of bonds.

To conclude, we have shown that the energy or force bottleneck, the path of least resistance to fracture, provides an early prediction of the path of the mode I crack that leads to failure in heterogeneous quasibrittle granular materials like concrete. The relationships between the flow bottlenecks and the various paths determined from key fracture criteria (viz. Minimum strain energy density, Maximum energy release rate and Maximum normal (Rankine) stress) highlight the salient influences of disorder in the bond network, heterogeneities in bond properties and stage of loading on the ultimate crack path. Building on recent work^24–30^, a direct comparison between flow bottlenecks and crack paths from experiments with more complex loading conditions (e.g., 3-point and 4-point bending tests) as well as cracking mechanisms in other materials like clay is now the subject of an ongoing investigation. Upscaling the method from laboratory to field is also being explored using a wide range of data sets, including proxies for force and energy^19,20^, with a view toward developing tools for practical decision-making, especially in material design and in Early Warning Systems (EWS) for failure hazard monitoring in natural and man-made structures^44^.

## Input data and DEM simulations

Data for the virtual samples D1–D3, all submitted to uniaxial tension, came from a family of discrete element (DEM) models of fracture in concrete (D2) and other granular composites (D1, D3)^24–30,35^. These models comprise planar (1 grain thick) and fully 3D samples for 2-phase cement composites (aggregate, cement matrix), 3-phase concrete (aggregate, cement matrix, interfacial transitional zones (ITZs)), using the explicit 3D spherical, open-source DEM code YADE^45,46^. The performance of these models for describing fracture, fracture characteristics and size effect for real quasibrittle cement composites, with particular attention paid to ordinary concrete, has been assessed under different experimental loading conditions: bending^25,28^, uniaxial compression^26,35^ and splitting tension^27^. Good agreement between numerical and experimental results on real concrete was achieved.

**Figure 11:**
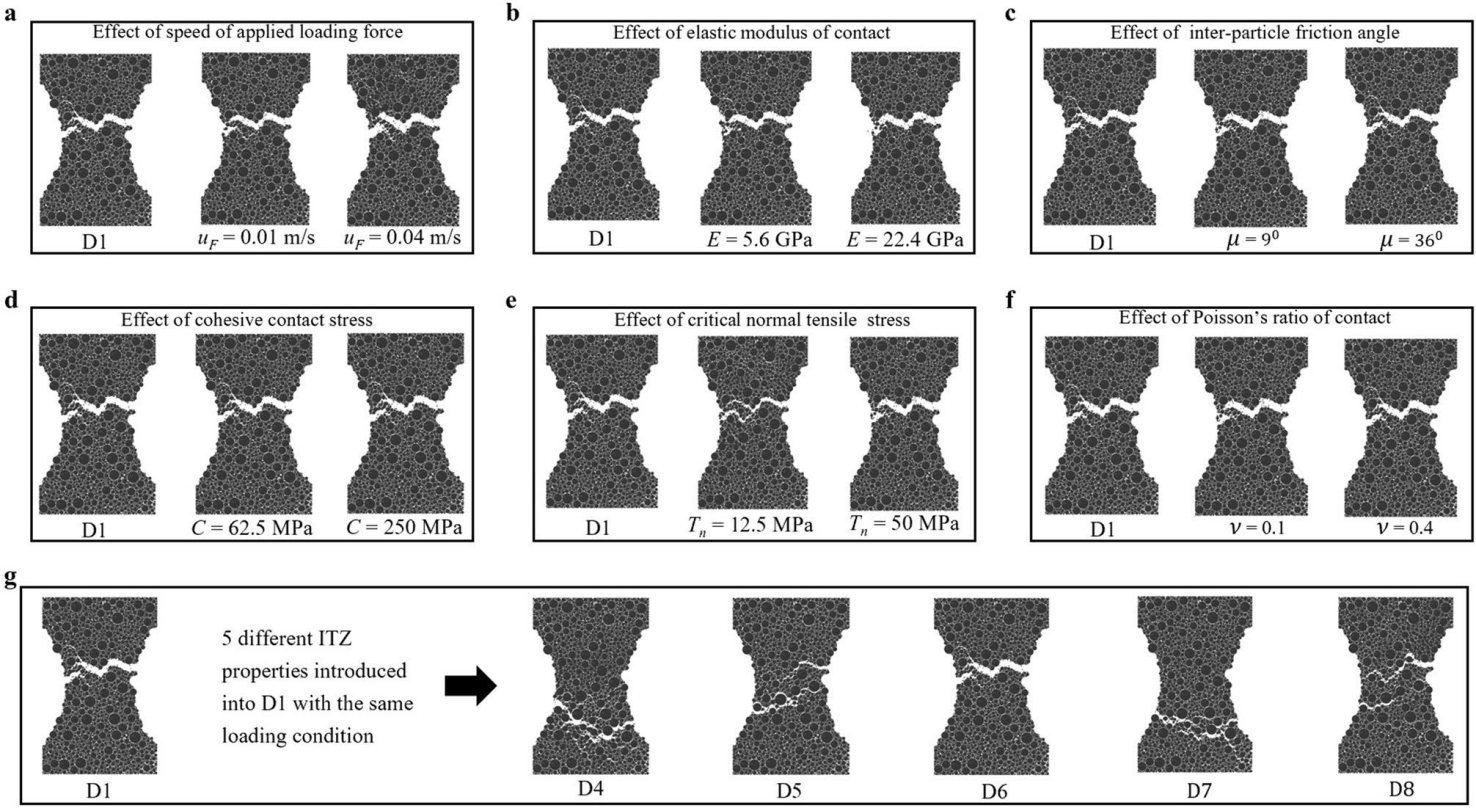
(Color online) Influence of various fracture properties and loading conditions on the crack geometry and location with D1 as the reference specimen: (**a**–**f**) without ITZ, (**g**) with ITZ. A list of parameter values for samples D1–D8 is given in Table 1.

D1 is from a “dog-bone” shaped specimen (2-phase): 150 mm high and 100 mm wide (60 mm at the mid-height). It consists of 4942 spherical grains: 704 aggregate grains (diameter range of 2–10 mm) and 4238 cement matrix grains (diameter range of 0.5–2 mm). D2 is from a rectangular concrete specimen: 150 mm high and 100 mm wide with two diagonally opposite U-shaped notches. Each notch is of size 15 mm $\times$ 5 mm: the notch on the left (right) boundary is 50 mm (100 mm) from the bottom boundary. The specimen is modeled as a 3-phase material composed of aggregate, cement matrix and ITZs. Aggregate grains possess ITZs which are simulated as contacts between aggregate and cement matrix grains. The cement matrix grains have no ITZs. The ITZs are weaker by 30% than the cement matrix. There are 200 aggregate spherical grains (diameter range of 2–16 mm) and 8000 cement matrix spherical grains (diameter range of 0.25–2 mm). The 3D specimen D3, here 2-phase for simplicity, is 100 mm high, 100 mm wide (60 mm at the mid-height) and 100 mm deep. There are 44,386 aggregate spherical grains (diameter range 2–10 mm) and 199,148 cement matrix spherical grains (diameter range of 0.75–2 mm). Overall, the grains comprise $95\%$ of the specimen in D1, D2 and D3. All the material parameters were calibrated against the experiment of van Mier^47^ (Table 1).

We investigated the influence of the different parameters and ITZ properties on the macrocrack geometry and location in prior studies^26,30^ (see also the Supplementary file). For completeness, a brief summary of the key findings is given in Fig. 11 using D1 as the reference sample. As shown, the DEM parameters, *viz.*, loading speed $u_F$, Poisson’s ratio $\nu$, inter-particle friction angle $\mu$ and cohesive contact stress *C*, have little to no influence on the macrocrack trajectory under uniaxial tension test conditions (Fig. 11a–f). By contrast, the presence of ITZs has a significant influence on the fracture pattern (Fig.11g). Note that in our past experimental studies of ordinary concrete, we observed ITZs on the concrete surface by means of scanning electron microscope (SEM) and nano-indentation tests^30,48,49^. They exist adjacent to aggregates and have a width of about 20–100 $\mu$m; when compared to the cement matrix, they reveal pronounced compositional differences which are strongest next to the aggregate surface and gradually diminish with distance away from the aggregate interface, though negligible beyond 15–100$\upmu$m. Although debate continues about the properties and mechanical effects of ITZs^11,50,51^, we found these to comprise more and larger pores, smaller particles, and a reduced stiffness and strength relative to the bulk phase^27,29^. Thus, they cannot be ignored in the DEM simulations.

**Table 1:** Virtual DEM samples and their corresponding parameters.

Sample	Parameters
D1	$u_F$ = 0.02 m/s, *E* = 11.2 GPa, $T_n$ = 25 MPa, *C* = 125 MPa, $\nu$ = 0.2, $\mu = 18^{\circ }$
D2	$u_F$ = 0.02 m/s, $E^{CM}$ = 11.2 GPa, $E^{ITZ}$ = 4.48 GPa, $T_n^{CM}$ = 24.5 MPa, $T_n^{ITZ}$ = 17.5 MPa, $C^{CM}$ = 125 MPa, $C^{ITZ}$ = 90 MPa, $\nu ^{ITZ} = \nu ^{CM}$ = 0.2, $\mu ^{ITZ} = \mu ^{CM} = 18^{\circ }$
D3	$u_F$ = 0.02 m/s, *E* = 11.2 GPa, $T_n$ = 24.5 MPa, *C* = 125 MPa, $\nu$ = 0.2, $\mu = 18^{\circ }$
D4-D6	$u_F$ = 0.02 m/s & $(E^{ITZ},T_n^{ITZ},C^{ITZ},\nu ^{ITZ},\mu ^{ITZ}) = h(E^{CM},T_n^{CM},C^{CM},\nu ^{CM},\mu ^{CM})$, where *h* = 0.5 for D4, *h* = 0.75 for D5, *h* = 1 for D6, $E^{CM}$ = 11.2 GPa, $T_n^{CM}$ = 25 MPa, $C^{CM}$ = 125 MPa, $\nu ^{CM}$ = 0.2, $\mu ^{CM} = 18^{\circ }$
D7	The parameters are the same as that for D6 except $T_n^{ITZ}$ = 18.75 MPa
D8	The parameters are the same as that for D6 except $T_n^{ITZ}$ = 12.5 MPa

## Supplementary information

Supplementary Information.

## Data Availability

The data in this paper are available upon reasonable request to Michał Nitka and Jacek Tejchman.

## List of symbols

$\alpha (e)$: Bond capacity of link *e*
$\alpha _E(e)$: Bond capacity for energy of link *e*
$\alpha _F(e)$: Bond capacity for force of link *e*
$\bar{\alpha }$: Residual bond capacity
$\bar{\alpha }_{E}(\epsilon )$: Residual bond energy
$\Gamma$: An arbitrary, feasible crack path (cut) in ${\mathcal {N}}$
$\Gamma _{min}$: Minimum cut in ${\mathcal {N}}$
$\Gamma _{max}$: Maximum cut in ${\mathcal {N}}$
$\gamma (e)$: Griffith’s fracture surface energy of link *e*
$\gamma (\Gamma )$: Griffith’s fracture surface energy for path (cut) $\Gamma$
$\hat{\gamma }(\Gamma )$: Griffith’s fracture surface energy density for path $\Gamma$
$\nu$: Poisson’s ratio of contact
$\nu ^{ITZ}$: Poisson’s ratio of cement-ITZ or ITZ-ITZ contacts
$\nu ^{CM}$: Poisson’s ratio of cement-cement contacts
$\mu$: Inter-particle friction angle
$\mu ^{ITZ}$: Inter-particle friction angle for cement-ITZ or ITZ-ITZ contacts
$\mu ^{CM}$: Inter-particle friction angle for cement-cement contacts
$\delta ^+(v)$: Set of arcs emanating from *v*
$\delta ^-(v)$: Set of arcs terminating at *v*
$$\epsilon$$: Time stage
$\Theta (\Gamma )$: Energy release rate at $\Gamma$
$$\phi$$: Flow in $$\mathcal {F}$$
$\phi (e)$: Flow on arc *e*
$\Phi (\Gamma )$: Total flow through $\Gamma$
$\Phi (\{s\})$: Net flow leaving the source node *s*
$\Phi _{max}$: Maximum flow from *s* to *t* in $\mathcal {F}$
*A*: Set of arcs
${\mathcal {C}}$: Actual path of the ultimate macrocrack
$B(\mathcal {F}_E)$: Bottleneck of the energy flow network $\mathcal {F}_E$
$B(\mathcal {F}_F)$: Bottleneck of the force flow network $\mathcal {F}_F$
$B(\bar{\mathcal {F}_E})$: Bottleneck of the residual energy flow network $\bar{\mathcal {F}_E}$
$B(\bar{\mathcal {F}_F})$: Bottleneck of the residual force flow network $\bar{\mathcal {F}_E}$
*b*: Bond area
*c*: Capacity function
$c(B(\mathcal {F}))$: Capacity of the bottleneck $B(\mathcal {F})$
*C*: Cohesive contact stress
$C^{ITZ}$: Cohesive contact stress for cement-ITZ or ITZ-ITZ contacts
$C^{CM}$: Cohesive contact stress for cement-cement contacts
*E*: Young’s elastic modulus
$E_{res}(\Gamma )$: Residual capacity of $\Gamma$
$E_{strain}(\mathcal{N} \setminus \Gamma , \epsilon )$: Sum of strain energies stored at all the contacts in $\mathcal{N}$ excluding those in the virtual path (cut) $\Gamma$ at loading stage $\epsilon$
$E_{tot}(\Gamma , \epsilon )$: Total energy of $\mathcal{N}$ at loading stage $\epsilon$
*e*: Link in *E* corresponding to a bond in ${\mathcal {N}}$
$$\mathcal {F}$$: Flow network
$\mathcal {F}_1$: Flow network with unit capacity function
$\mathcal {F}_E$: Energy flow network
$\bar{\mathcal {F}}$: Residual flow network
$\mathcal {F}_F$: Force flow network
$\bar{\mathcal {F}_E}$: Residual energy flow network
$\bar{\mathcal {F}_F}$: Residual force flow network
$f(e,\epsilon )$: Realized flow (bond flow) of link *e* at loading stage $\epsilon$
$f_E(e,\epsilon )$: Realized energy flow (bond energy) of link *e* at loading stage $\epsilon$
$f_F(e,\epsilon )$: Realized force flow (bond force) of link *e* at loading stage $\epsilon$
$F_{min}^n(e)$: Fracture strength of bond or link *e*
$\mathbf {F}_n$: Tensile normal force
*G*: Directed network
$K_n(e)$: Normal stiffness of link *e*
*m*: Final time stage of loading
${\mathcal {N}}$: Bond contact network
$\mathcal {P}$: Optimized flow routes
*P*: Percentage error of prediction
$$p_{min}$$: Pathway redundancy
*r*: Radius of the smaller of the two grains sharing the bond
$r_A, r_B \ \ (r=r_A \le r_B)$: Radii of the two grains sharing the bond
$\mathbb {R}_0^+$: Non-negative real numbers
$S(\Gamma )$: Edges in ${\mathcal {N}}$ that are saturated
*s*: Source node
*t*: Sink node
$T_n(e)$: Critical normal tensile stress of bond or link *e*
$T_n^{ITZ}$: Critical normal tensile stress for cement-ITZ or ITZ-ITZ contacts
$T_n^{CM}$: Critical normal tensile stress for cement-cement contacts
$U(e,\epsilon )$: Potential energy of bond or link *e* at loading stage $\epsilon$
$u_F$: Speed of applied loading force
*V*: Set of nodes
*v*: Node
$V_{B_u}$: Set of grains in the upper part of the bottleneck
$V_{{\mathcal {C}}_u}$: Set of grains in the upper part of the macrocrack
$W,W'$: Two distinct node sets of *V*
$V_T$: All the grains in the sample
