# Supplementary material for: Early prediction of macrocrack location in concrete, rocks and other granular composite materials
Source: Sci Rep. 2020 Nov 20;10:20268. doi: 10.1038/s41598-020-76616-y (PMC7679422; doi:10.1038/s41598-020-76616-y)
Supplement: Supplementary file 1 — Supplementary Information. [file 41598_2020_76616_MOESM1_ESM.doc]

**SUPPLEMENTARY DISSCUSSIONS, DATA AND REFERENCES**

**Early prediction of macrocrack location in concrete, rocks and other granular composite materials**

Antoinette Tordesillas^1,*^, Sanath Kahagalage^1^, Michał Nitka^2^, and Jacek Tejchman^2^

^1^The University of Melbourne, School of Mathematics & Statistics, Melbourne, 3010, Australia

^2^Gdansk University of Technology, Faculty of Civil & Environmental Engineering, Narutowicza 11/12, 80-233 Gdansk, Poland

^*^**Correspondence** should be addressed to A.T. (atordesi@unimelb.edu.au)

**Additional information on DEM simulation**

The capability of our DEM model to simulate the concrete behavior (described as 3/4-phase material) was successfully validated in uniaxial tension and uniaxial compression in 2D/3D conditions [1], [2], in 2D splitting test [3], [4] and 2D/3D bending beam tests [5]-[8]. A satisfactory agreement was achieved between experiments and DEM analyses with respect to stress-strain/force-displacement curves and fracture pattern.

The interfacial transition zones (ITZs) are weak porous zones around aggregates in usual concretes with a width of about 20-100 µm. It is very difficult to describe the mechanical properties of ITZs. Their stiffness can be determined with the aid of nanoindentation tests, however, their strength not. The parameters needed in DEM calculations must take the stiffness and strength of ITZs into account. Therefore, we always calibrate DEM in a classical way by comparing the DEM simulation results with simple laboratory test outcomes with respect to the stress-strain curve (stiffness, strength and brittleness) and fracture pattern.

The bond (contact) between particles is brittle in tension (see Figure S1, tension is below a horizontal axis). After the minimum normal tensile force *F_n_^min^ = T_n_×r^2^* is reached, the bond simply brakes, and the contact disappears. These parameters are calibrated against the experiments and this procedure in detail can be found in [1], [5]. This is a commonly used formula for the bond strength ([9]-[11]). A softening regime was not assumed beyond the force *F_n_^min^*. Hence, the local fracture energy (that characterizes a softening branch of stress-strain or force-displacement curves) was equal to zero. The global softening solely comes in DEM from the bonds’ breakage. Note that DEM models for soils do not also possess softening.


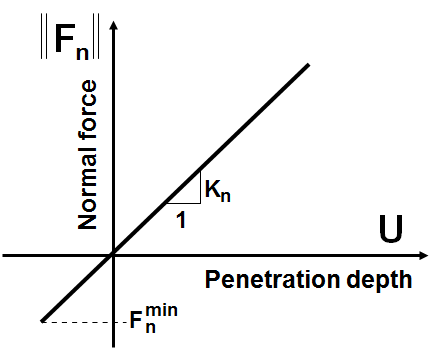


Figure S1: Normal contact law (when normal contact force *F_n_* reaches *F_n_^min^*, bonded contact breaks).

The normal contact force is given by *F_n_*=*K_n_* *U* [2] and elastic energy is given by 0.5*K_n_ U*^2^ = 0.5 *F_n_*^2^/*K_n_*. Therefore, the maximum elastic energy is given by *E_max_*=0.5 (*F_n_^min^*)2/*K_n_*. Thus, when the elastic strain energy reaches *E_max_*, bonds break [4].

**Number of ITZ contacts in the bottleneck of the sample D2**

The existence of very porous heterogeneous ITZs in concrete around aggregate particles is crucial from the point of view of mechanics since they are usually the weakest zones in ordinary concretes due to their significant porosity and reduced stiffness and strength. Thus, they become attractors for macro-cracks as weak links - they offer an easier pathway for the crack growth that gradually evolve by bridging interfacial micro-cracks [6], [7]. Thus, they cannot be simply omitted since they govern global concrete properties (strength, brittleness and fracture). Indeed, we observed that more than 60% of ITZ contacts in the bottleneck of D2 (Figure S2).


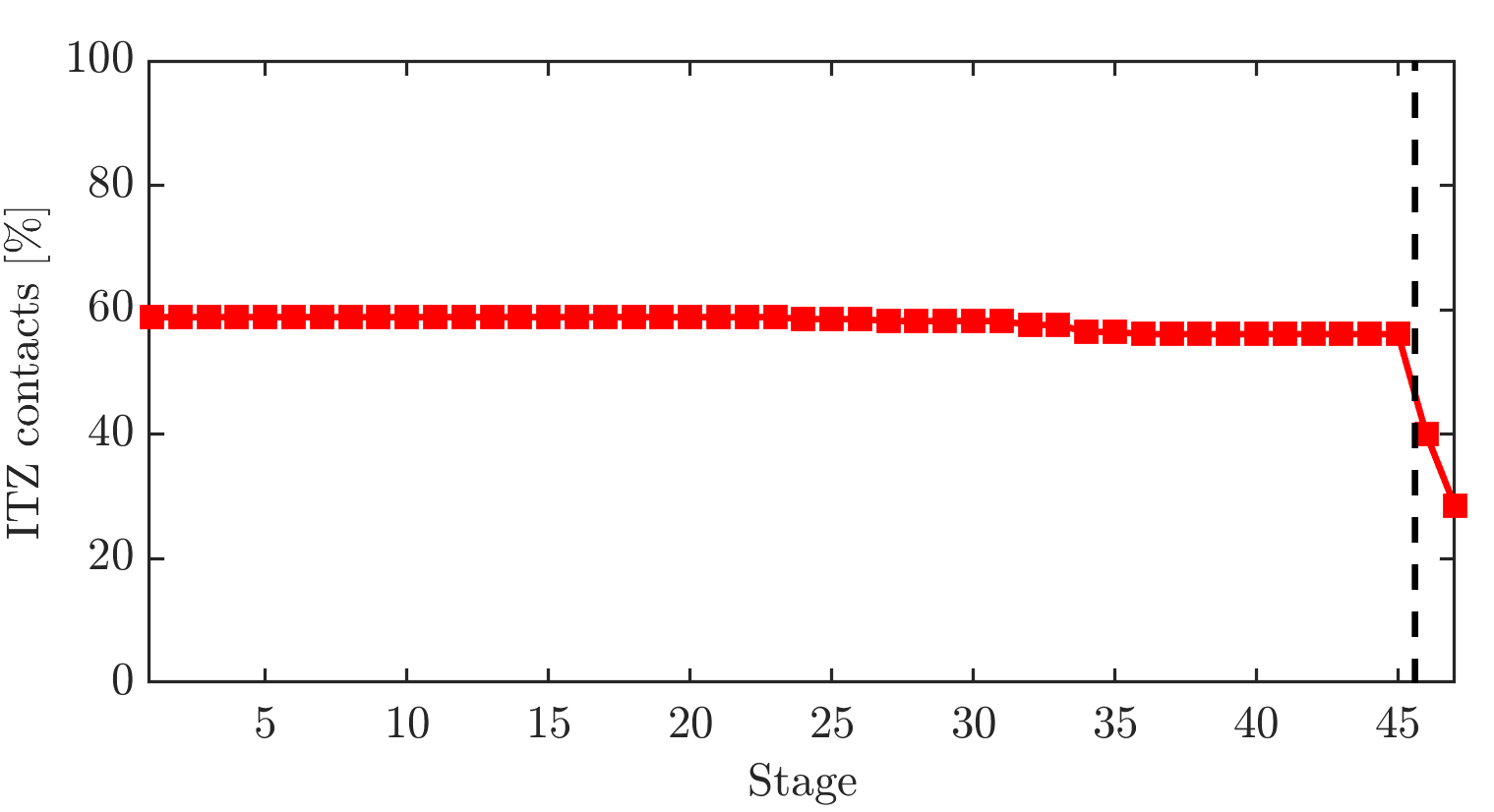


Figure S2: The evolution of stage with the percentage of ITZ contacts in the bottleneck of the sample D2.

**Effects of parameters on the ultimate macro-crack path**

In our calculations for uniaxial tension, we showed that the macro-crack’s geometry depends on: internal meso-structure (size, shape and location of aggregates, volume of micro-pores) and the stiffness and strength of ITZs. The influence of some different parameters on the macro-crack’s path was investigated and shown in Figure S3. For concrete as a 2-phase material, the macro-crack path was slightly dependent upon the parameters *T_n_* and *E*. The rest of the parameters (loading velocity *u_F_*, the Poisson’s coefficient *ν*, inter-particle friction angle *µ* and cohesive contact strength *C*) had no impact on the macro-crack trajectory. The presence of weak ITZs around aggregates (concrete described as a 3-phase material) obviously affects the shape of the macro-crack (mostly the parameter *T_n_^ITZ^*).

Table S1: Virtual DEM samples in Figure S3 and their corresponding parameters.


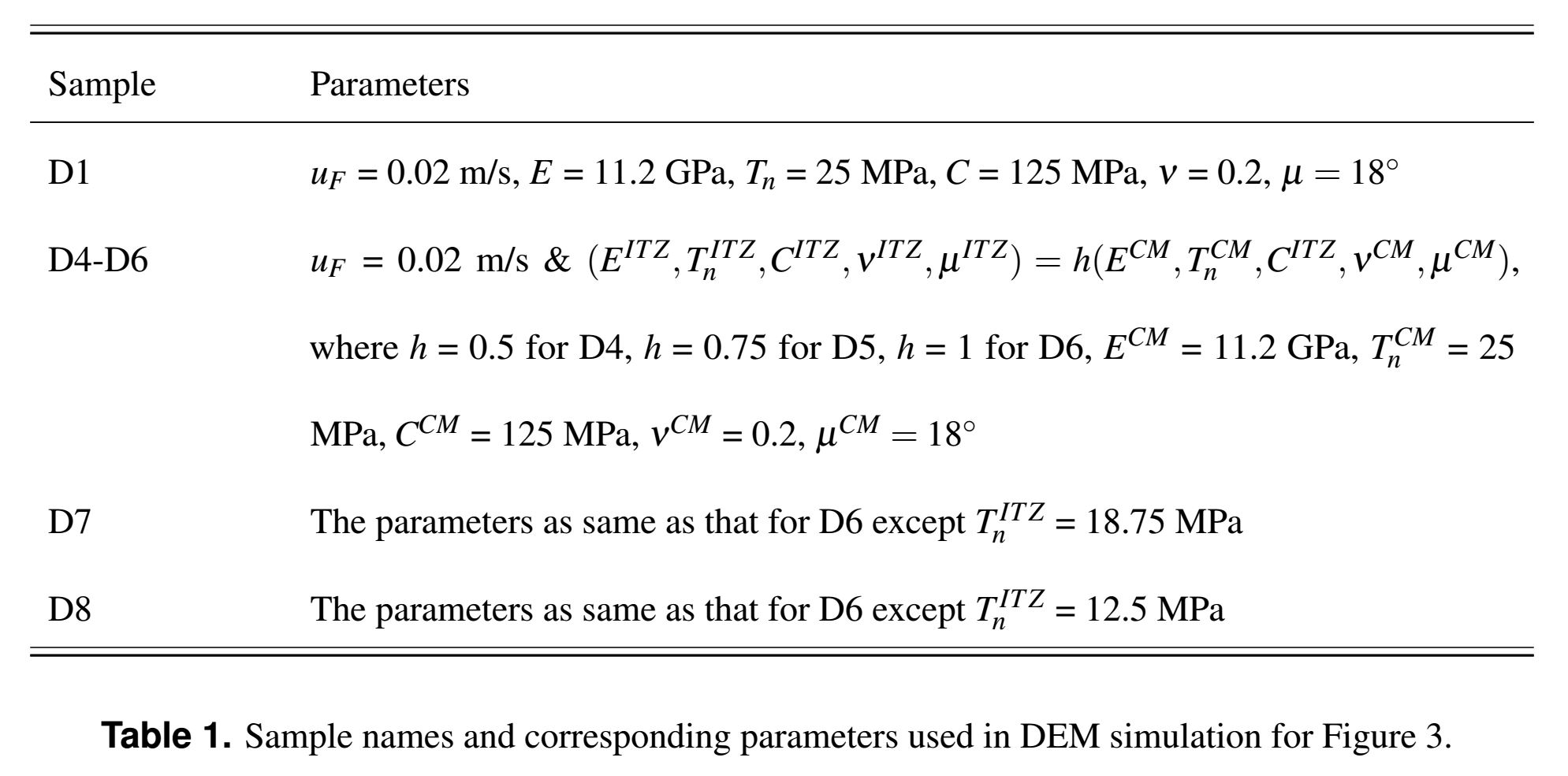


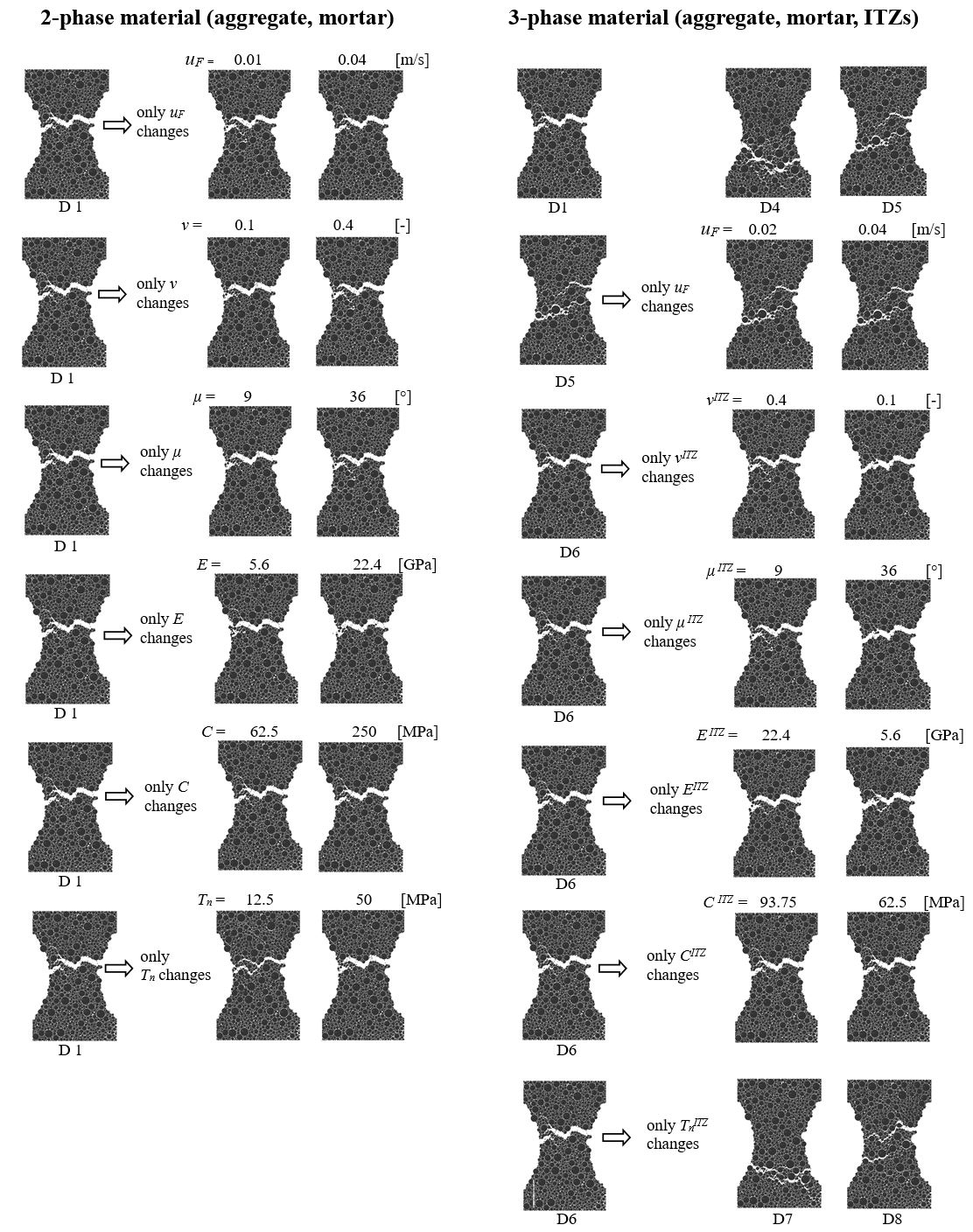


Figure S3: Effects of parameters on the ultimate macro-crack path. The parameters are given in Table S1.

**References**

1. [1] Nitka M, Tejchman J. (2015) Modelling of concrete behaviour in uniaxial compression and tension with DEM. *Granular Matter* **17**(1), 145-164.
2. [2] Suchorzewski J., Tejchman J., Nitka M. (2018) Discrete element method simulations of fracture in concrete under uniaxial compression based on its real internal structure. *International Journal of Damage Mechanics* **27**, 578-607.

[3] Suchorzewski J., Tejchman J., Nitka M. (2018) Experimental and numerical investigations of concrete behaviour at meso-level during quasi-static splitting tension. *Theoretical and Applied Fracture Mechanics* **96**, 720-739.

1. [4] Suchorzewski J., Tejchman J., Nitka M., Bobiński J. (2019) Meso‐scale analyses of size effect in brittle materials using DEM. *Granular Matter* **21** (9), 1-19.
2. [5] Skarżyński Ł., Nitka M., Tejchman J. (2015) Modelling of concrete fracture at aggregate level using FEM and DEM based on X-ray CT images of internal structure. *Engineering Fracture Mechanics* **147**, 13-35.
3. [6] Nitka M., Tejchman J. (2018) A three-dimensional meso-scale approach to concrete fracture based on combined DEM with x-ray micro-CT images. *Cement and Concrete Research* **107**, 11-29.
4. [7] Nitka M, Tejchman J. (2020) Meso-mechanical modelling of damage in concrete using discrete element method with porous ITZs of defined width around aggregates. *Engineering Fracture Mechanics* **231**, 107029.
5. [8] Nitka M., Tejchman J. (2020) Comparative DEM calculations of fracture process in concrete considering real angular and artificial spherical aggregates. *Engineering Fracture Mechanics* **239**. 107309, <https://doi.org/10.1016/j.engfracmech.2020.107309>
6. [9] Ergenzinger C., Seifried R., Eberhard P. A. (2011) Discrete Element Model to Describe Failure of Strong Rock in Uniaxial Compression. *Granular Matter* **13**(4):341-364.
7. [10] Donze F. V., Magnier S. A., Daudeville L., Mariotti C. (1999) Numerical study of compressive behaviour of concrete at high strain rates. *Journal for Engineering Mechanics* **122**(80):1154-1163.
8. [11] Scholtes, L. and Donze, F-V. (2013). A DEM model for soft and hard rocks: role of grain interlocking on strength. *Journal of the Mechanics and Physics of Solids*. **61**(2): 352-369.
